# Insights into the Role of Oxidative Stress in Ovarian Cancer

**DOI:** 10.1155/2021/8388258

**Published:** 2021-10-07

**Authors:** Dan-Ni Ding, Liang-Zhen Xie, Ying Shen, Jia Li, Ying Guo, Yang Fu, Fang-Yuan Liu, Feng-Juan Han

**Affiliations:** ^1^Department of Obstetrics and Gynecology, Heilongjiang University of Chinese Medicine, Harbin 150040, China; ^2^Department of Obstetrics and Gynecology, The First Affiliated Hospital of Heilongjiang University of Chinese Medicine, Harbin 150040, China

## Abstract

Oxidative stress (OS) arises when the body is subjected to harmful endogenous or exogenous factors that overwhelm the antioxidant system. There is increasing evidence that OS is involved in a number of diseases, including ovarian cancer (OC). OC is the most lethal gynecological malignancy, and risk factors include genetic factors, age, infertility, nulliparity, microbial infections, obesity, smoking, etc. OS can promote the proliferation, metastasis, and therapy resistance of OC, while high levels of OS have cytotoxic effects and induce apoptosis in OC cells. This review focuses on the relationship between OS and the development of OC from four aspects: genetic alterations, signaling pathways, transcription factors, and the tumor microenvironment. Furthermore, strategies to target aberrant OS in OC are summarized and discussed, with a view to providing new ideas for clinical treatment.

## 1. Introduction

Ovarian cancer (OC) is the third most common cancer among women, with 290,000 women diagnosed and 180,000 dying every year globally, and OC is the most lethal gynecological cancer with a five-year survival below 45% [1, 2]. Currently, OC is divided into type I and type II tumors according to the clinical, genetic, and histopathological factors in the revised ovarian carcinogenesis model [3]. Type I tumors develop from benign extraovarian precursor lesions that are present in the ovary, and these tumors are mostly confined to the ovary and have a good prognosis, accounting for only 10% of OC-related deaths. Type II tumors are generally thought to develop from serous tubal intraepithelial carcinoma based on both shared TP53 mutations and integrated molecular analyses [4]. They are more aggressive, resulting in 90% of the deaths from OC, of which high-grade serous carcinoma (HGSC) is by far the most common form of diagnosis, resulting in 70–80% of deaths from OC [5, 6]. Understanding the etiology of OC is of great significance for its prevention and treatment. The current treatment for OC includes surgical resection, chemotherapy, neoadjuvant chemotherapy, radiotherapy, and immunotherapy [7, 8]. However, more than 50% of OC patients eventually relapse and suffer from late metastasis, and secondary adverse reactions and drug resistance pose major challenges to treating OC [9].

**Figure 1:**
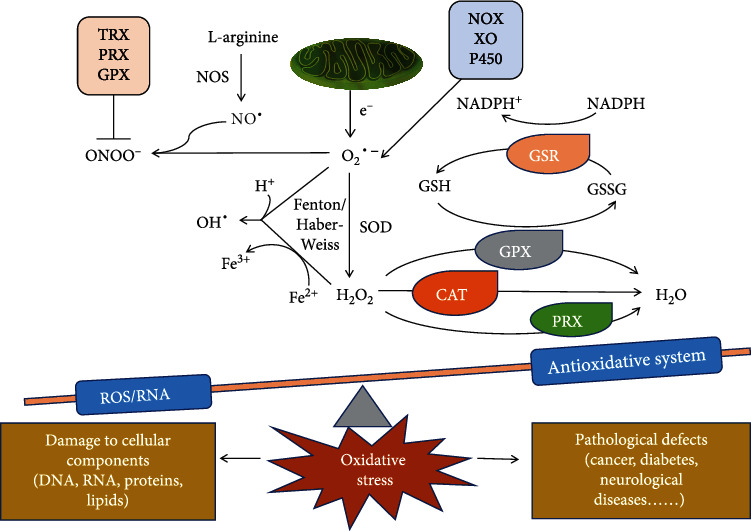
The major oxidative and antioxidant systems. Both electron leakage from the mitochondria and enzymatic activity of the oxidase system, as represented by NOX, XO, and cytochrome P450, produce O_2_·^–^. SOD converts O_2_·^–^ into H_2_O_2_ [233], and in the presence of reducing transition metals, such as ferrous ions, H_2_O_2_ is converted into highly active OH· by the Fenton or Haber-Weiss reaction [26]. H_2_O_2_ is converted into H_2_O by CAT, PRX, and GPX. In the GPX reaction, GSH is oxidized to GSSG (glutathione disulfide), which can be converted back to GSH by GSR during NADPH consumption [15]. L-Arginine is converted to NO· under the catalysis of nitric oxide synthase (NOS), which reacts with O_2_·^–^ to form ONOO^−^ [234]. TRX, PRX, and GPX can inhibit ONOO^−^ generation [24]. Each ROS has different physical and chemical properties and half-lives. Among these, OH· has the strongest oxidizing property, followed by O_2_·^–^, while H_2_O_2_ is relatively weak. H_2_O_2_ and NO· also play essential roles as signaling molecules [31].

Oxidative stress (OS) arises when there is an imbalance between the production of free radicals and reactive metabolites (so-called prooxidants, including reactive oxygen and nitrogen species) and intrinsic antioxidant defenses. This imbalance leads to damage of biological molecules and tissues and has the potential to impact on the whole organism [10, 11]. Reactive oxygen species (ROS) are represented by free radicals and nonfree radical oxygen-containing molecules, including superoxide anions (O_2_·^–^), hydrogen peroxides (H_2_O_2_), singlet oxygens (^1^O_2_), and hydroxyl radicals (OH·) [12, 13]. Reactive nitrogen species (RNS) include nitric oxide (NO·) and peroxynitrite anions (ONOO^–^) [14] (Figure 1). The generation of ROS and RNS is dependent on both enzymatic and nonenzymatic reactions. Enzymatic reactions mainly involve nicotinamide adenine dinucleotide (NADPH) oxidase (NOX), xanthine oxidase (XO), peroxidase, and the cytochrome P450 system [15–17], while nonenzymatic reactions, i.e., electron leakage from the mitochondrial respiratory chain, are the main source of ROS [18]. To avoid OS, cells possess a series of nonenzymatic and enzymatic antioxidant defense systems. The nonenzymatic defense system includes flavonoids, vitamins (A, C, and E), and glutathione (GSH) [19, 20], while the enzymatic antioxidant system consists of superoxide dismutase (SOD), catalase (CAT), glutathione peroxidase (GPX), glutathione reductase (GSR), glutathione S-transferases (GST), peroxiredoxin (PRX), and thioredoxin (TRX) [21–25] (Figure 1). In healthy organisms, ROS and RNS are normal by-products of cellular metabolism and participate in the transduction of different signaling pathways, and the antioxidant defense system will quickly remove them before they cause damage to cellular structure and function [22, 26]. However, many endogenous factors (mental stress, inflammation, various enzymatic systems, etc.) and exogenous factors (ultraviolet radiation, ionizing radiation, air pollution, etc.) can induce excessive production of ROS and RNS in cells [27–29]. This heightened production of oxidants can overwhelm the body's antioxidant defense system leading to a state of OS, which in turn leads to irreversible oxidative damage to proteins, lipids, and nucleic acids [30]. Such damage interferes with key cellular functions, and this is closely related to the occurrence and development of cancer, diabetes, and cardiovascular and nervous system diseases, to name just a few [31, 32] (Figure 1).

OS has been widely confirmed to play a dual role in the pathogenesis of cancer [11, 33]. On the one hand, ROS/RNS can promote molecular genetic changes that lead to tumor initiation, growth, and development as well as to therapeutic resistance [34, 35]. On the other hand, long-term elevated ROS/RNS levels have cytotoxic effects and can induce the activation of apoptotic pathways [35, 36]. It is well known that cancer cells contain higher levels of reactive molecules that maintain the cellular phenotype and high proliferation rate, and tumor cells must find ways to optimize ROS-driven proliferation while avoiding ROS thresholds that trigger senescence, apoptosis, or ferroptosis [37, 38]. Several studies on OC have confirmed this and have shown upregulated responses to OS in OC cells. For example, NOX, inducible nitric oxide synthase (iNOS) [39–41], and SOD [42, 43] are overexpressed in OC compared with normal tissues, and GSTs have been reported to be overexpressed in human malignancies and to be associated with tumor progression and drug resistance [44]. OS triggers cancer cells to produce lactic acid by glycolysis rather than oxidative phosphorylation, even under aerobic conditions, which is referred to as aerobic glycolysis or the Warburg effect [45]. The Warburg effect has been used to detect and monitor tumor progression in the clinic using positron emission tomography to detect highly glycolytic regions in the body by measuring 2-[^18^F] fluoro-2-deoxy-D-glucose (a glucose analogue concentrated in highly proliferative tumorigenic tissues) [46]. Iron and its metabolites promote the generation of ROS via the Fenton reaction, and these ROS induce DNA damage thus leading to carcinogenesis. For example, when chocolate cysts form, the ovary contains old blood with excessive iron, which may increase the risk of malignant transformation of endometriosis into ovarian clear cell carcinomas [47]. At the same time, when cells are moderately exposed to nontoxic doses of H_2_O_2_, glucose-6-phosphate dehydrogenase (G6PD) is activated, which leads to a switch in glucose metabolism from glycolysis to the pentose phosphate pathway (PPP) and nucleotide synthesis. This approach avoids exposing tumor cells to OS due to the production of ROS and provides a survival advantage for the development of cancer [48, 49]. In addition, NADPH produced by the PPP is used for reductive biosynthetic reactions in cells, which enhances the GSH and TRX-based antioxidant systems and maintains ROS levels in a state of dynamic equilibrium [50–53]. Cells adapt to OS through metabolic reprogramming in the short term while genetic reprogramming provides long-term adaptation [54–56]. OS can regulate the redox state of tumor cells by regulating gene expression or activating different transcription factors, such as activating protein 1, HIF-1*α*, heat shock factor 1, NF-*κ*B, Nrf2, and the tumor inhibitor p53 [11, 57]. Additionally, ROS also interact with signaling molecules such as mitogen-activated protein kinase (MAPK), phosphatidylinositol 3-kinase (PI3K), phosphatase and tensin homolog (PTEN), and protein tyrosine phosphatase (PTP) by targeting reactive cysteine residues in proteins to activate a variety of cellular processes, thus regulating cell proliferation, differentiation, migration, and apoptosis [58–61].

Many factors are involved in OC, including genetic factors, age, infertility, nulliparity, microbial infections, obesity, and smoking [7, 8]. A number of studies have been conducted on the role of OS in the development of OC [16, 62–66], and the strong association between OS and OC is gradually becoming a hot topic of interest. This review updates the relevant literature and focuses on teasing out the effects of OS on OC from different perspectives. In addition, we comprehensively analyze the interaction between OS and genetic alterations, different signaling pathways and transcription factors, and different components of the tumor microenvironment (TME) to further explore the role of OS in the pathogenesis and treatment of OC.

## 2. OS-Related Pathogenesis in OC

**Figure 2:**
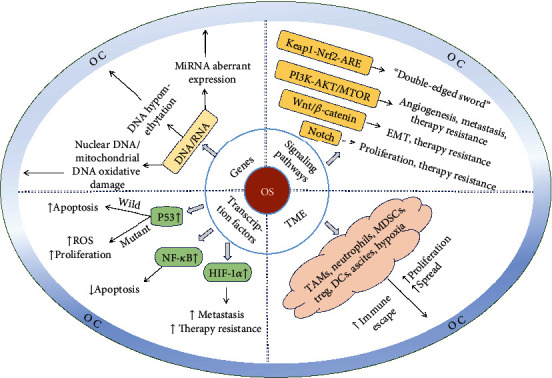
OS-related pathogenesis in OC.

OS leads to the development of OC through four aspects: genetic alterations, signaling pathways, transcription factors, and the TME (Figure 2).

### 2.1. OS-Mediated Genetic Alterations in OC

OC can be triggered through OS-mediated genetic alterations such as oxidative damage of nuclear DNA and mitochondrial DNA (mtDNA), DNA hypomethylation, and abnormal expression of microRNA. The specific mechanisms are as follows.

DNA damage and genetic instability caused by OS play a crucial role in the occurrence and development of OC [67, 68]. H_2_O_2_ and the hydroxyl radicals generated by the Fenton reaction have been shown to promote the transferrin- (Tf-) TfR1 axis, which induces DNA double-strand breaks in epithelial cells of the fallopian tube, which promotes the progression of OC [69]. The most common product of DNA oxidative damage is 8-hydroxy-2′-deoxyguanosine (8-OHdG), which is formed by guanine oxidation [70], and it is usually used as a biomarker of DNA damage as well as for assessing the risk associated with cancer progression [71–73]. In addition, 8-OHdG can induce C:G to A:T transformations, which are the most important somatic mutations in OC, breast cancer, lung cancer, and gastric cancer [74]. Also, about half of all patients with OC have abnormal homologous recombination repair (HRR) [8]. A recent study showed that inhibition or depletion of RAD51, a key protein involved in HRR, can lead to OS and increased DNA oxidative damage in OC, and this reflects the involvement of HRR in redox state regulation in OC [75].

mtDNA is more susceptible to oxidative damage than nuclear DNA because mitochondria house the electron transport chain that produces large amounts of ROS [76], and mitochondria lack effective DNA repair mechanisms [77]. mtDNA mutations have been reported in various cancers, including OC [78–81], and studies have shown that many chemical carcinogens preferentially bind to mtDNA rather than nuclear DNA [82]. In addition, damage to nuclear DNA can trigger mitochondrial reactions, while the increase in ROS in mitochondria can aggravate nuclear DNA damage [83, 84]; therefore, there may be a “vicious circle” between OS, DNA damage, and cancer development [85]. A correlation between mtDNA mutations and response to therapy in OC has also been reported. HGSC patients with heteroplasmic pathogenic mtDNA somatic mutations were found to have a higher incidence of platinum resistance and disease relapse compared to patients without pathogenic mtDNA somatic mutations (80% vs. 16.7%, *p* = 0.035), and the phenomenon of accumulation of oxidative damage-derived G>T and A>C somatic mutations in the tumor samples indicated that the tumor cells were exposed to OS [86]. Another study suggested that disruptive mtDNA mutations may be used as adjuvant prognostic molecular markers [87].

DNA methylation is one of the primary epigenetic mechanisms for regulating gene expression, and DNA hypomethylation has been reported to be associated with the initiation and progression of various cancers [88]. DNA methylation involves the covalent bonding of a methyl group to the 5th carbon position of the cytosine of the genomic CpG dinucleotide by DNA methyltransferase [89], and this process is inhibited by ROS, thus leading to DNA hypomethylation [90–92]. The change in DNA methylation is an early event in OC [93], and DNA hypomethylation has been shown to contribute to the high expression of SLC4A11 (a solute-linked cotransporter), which is related to the poor prognosis of OC caused by OS [94].

MicroRNAs (miRNAs) also play roles in the pathogenesis of OC [65, 95]. miRNAs are small noncoding RNAs that participate in the regulation of posttranscriptional gene expression by inducing mRNA degradation or inhibiting translation [96]. In addition, miRNAs are considered to be important mediators of the immune system, to be involved in inflammatory reactions, and to be closely related to the progress and treatment of diseases [97]. Several studies have demonstrated that OS can increase the sensitivity of OC cells to paclitaxel and promote the mesenchymal-epithelial transition by stimulating the overexpression of miR-141 and miR-200s [98, 99].

### 2.2. OS-Mediated Alterations of Signaling Pathways in OC

Many studies have confirmed the important role of redox modification of signaling pathways in the pathogenesis of OC, including the Keap1-Nrf2-ARE, PI3K/AKT/mTOR, Wnt/*β*-catenin, and Notch pathways [100–103].

#### 2.2.1. Keap1-Nrf2-ARE Signaling Pathway

**Figure 3:**
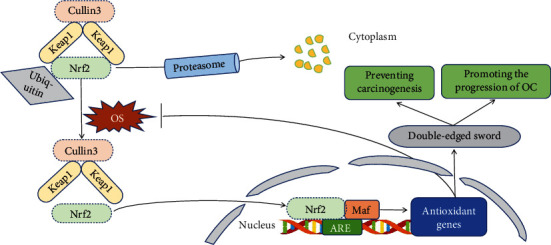
Transduction mechanism of the OS-mediated Keap1-Nrf2-ARE signaling pathway in OC. Under physiological conditions, Nrf2 is retained in the cytoplasm by Keap1. Keap1 binds Nrf2 to the Cullin3-dependent E3 ubiquitin ligase complex, which promotes the ubiquitination and subsequent proteasome degradation of Nrf2 [106, 107]. Under conditions of OS, the cysteine residues exposed on the surface of Keap1 are oxidized, which causes Nrf2 to dissociate from Keap1, translocate to the nucleus, form a heterodimer with Maf, and then bind with ARE, thereby transcriptionally activating Nrf2-regulated antioxidant gene expression and inhibiting OS [108, 109]. The activation of the Nrf2 pathway is a double-edged sword in OC, and it maintains the stability of the normal ovarian cell environment and genome in order to prevent OS-induced carcinogenesis [112], while it also protects tumor cells from OS thus enhancing the invasion and chemoresistance of OC [110, 115].

The Keap1-Nrf2-ARE signaling pathway is one of the most important pathways through which cells respond to OS, and it has attracted attention due to its ability to regulate a broad range of antioxidant and detoxification genes [31, 104]. Nuclear factor E2-related factor 2 (Nrf2), which is at the center of this pathway, regulates the transcription of genes that encode various detoxifying enzymes and antioxidant proteins [105]. Under homeostatic conditions, Nrf2 is usually localized in the cytoplasm, where it binds to the inhibitor Kelch-like ECH-associated protein 1 (Keap1). Keap1 is an adaptor protein for the Cullin3-dependent E3 ubiquitin ligase, which mediates the ubiquitination and subsequent degradation Nrf2 in order to maintain basal levels of the protein [106, 107] (Figure 3). When cells are exposed to OS, the cysteine residues of Keap1 are oxidized, which causes Nrf2 to dissociate from the Keap1 complex and translocate into the nucleus. There it forms a heterodimer with the Maf (musculoaponeurotic fibrosarcoma) protein, which then binds to ARE, the first antioxidant response element to be identified. Thus, Nrf2-regulated antioxidant gene transcription can be activated to exert an antioxidant effect [108, 109] (Figure 3). The activation of the Nrf2 pathway is considered to be a double-edged sword in OC [104, 110, 111] (Figure 3). On the one hand, the Nrf2 pathway can maintain the stability of the normal ovarian cell environment and genome to prevent carcinogenesis induced by OS [112]. On the other hand, the Nrf2 pathway protects OC cells from oxidative damage [113, 114] and helps them cope with various cytotoxic drugs, thus enhancing the invasive ability and chemotherapy resistance of OC [110, 115].

The chemotherapy resistance of OC cells is related to mutations within highly conserved domains of the *Keap1* gene [116] and to the activation of downstream genes of the Nrf2 pathway [117]. The downstream antioxidant proteins of the Nrf2 pathway mediate detoxification through glutathione coupling and participate in ATP-dependent drug efflux, which may be one of the mechanisms of drug resistance in OC cells [107]. In addition, inhibiting the production of proteasomes promotes the translocation of Nrf2 into the nucleus via the Keap1/Nrf2 pathway, resulting in drug resistance in OC cells. Nrf2 and peroxisome proliferator-activated receptor-*γ* coactivator 1*α* (PGC1*α*) can synergistically regulate antioxidant functions and mitochondrial functions, thus regulating the maintenance of proteasome activity and affecting the sensitivity of OC cells to chemotherapy agents [101].

Recently, the Keap1-Nrf2-ARE pathway has been shown to play an important role in the prognosis of OC. Cho et al. detected the expression of Nrf2 and Keap1 in 100 cases of epithelial ovarian cancer (EOC) tissues by immunohistochemistry and followed up all patients for a mean of 55.3 months. They found that Nrf2 was overexpressed in the cytoplasm of OC cells. Further survival analysis showed that high Keap1 expression predicted better overall survival and was an independent prognostic factor. Specifically, high levels of Keap1 in the cytoplasm can inhibit the nuclear translocation and enhance the negative feedback control of Nrf2, thereby inhibiting the survival of OC cells [118].

#### 2.2.2. PI3K/AKT/mTOR Signaling Pathway

**Figure 4:**
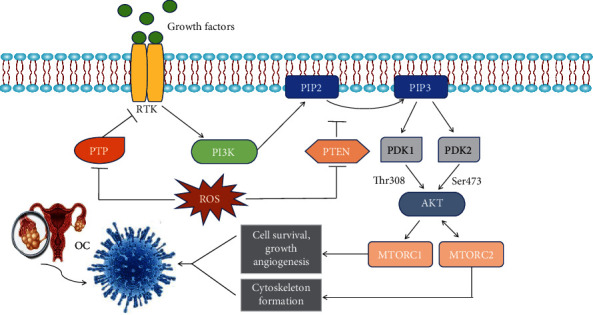
Transduction mechanism of the OS-mediated PI3K/AKT/mTOR signaling pathway in OC. Growth factors interact with receptor tyrosine kinase (RTK) leading to PI3K activation, which can be inhibited by PTP [136, 235]. Fully activated PI3K phosphorylates phosphatidylinositol bisphosphate (PIP2) to phosphatidylinositol trisphosphate (PIP3). This conversion results in the activation of AKT by phosphorylation of its kinase domain (Thr308) by PDK1 and phosphorylation of its C-terminal domain (Ser473) by PDK2. AKT can further activate mTOR, which includes mTOR complex 1 (mTORC1) and mTORC2. Activation of mTORC1 leads to cell survival, growth, and angiogenesis, while mTORC2 has been implicated in cytoskeleton formation and cell survival [235, 236]. PTEN reverses the effects of PI3K by dephosphorylating PIP3 [236]. ROS can inhibit the activity of PTP and PTEN by oxidizing cysteine residues, thus activating the PI3K/AKT/mTOR pathway [123–125] leading to the proliferation, migration, and chemotherapy resistance in OC.

The PI3K/protein kinase B/mammalian target of rapamycin (PI3K/AKT/mTOR) signaling pathway plays a central role in the proliferation, migration, and chemoresistance of OC [119–122], and the specific transduction mechanism is shown in Figure 4. ROS can inhibit the activity of PTP and PTEN by oxidizing cysteine residues, thus activating the PI3K/AKT/mTOR pathway [123–125] (Figure 4). Moreover, the expression of PTEN was reported to be decreased in 104 of 151 analyzed EOC samples [126]. OS facilitates the growth and metastasis of OC by activating the PI3K/AKT/mTOR pathway in order to increase the expression of vascular endothelial growth factor (VEGF) [127] and to promote de novo fatty acid and cholesterol biosynthesis [100]. In addition, NO can protect OC cells from apoptosis and can enhance drug resistance by activating the PI3K/AKT/mTOR pathway [128]. Interestingly, another study on OC showed a contrasting phenomenon in which ROS mediated apoptosis by inhibiting the PI3K/AKT/mTOR signaling pathway [129], which was related to the concentration of ROS and confirmed the dual role of OS in cancer.

#### 2.2.3. Wnt/*β*-Catenin Signaling Pathway

**Figure 5:**
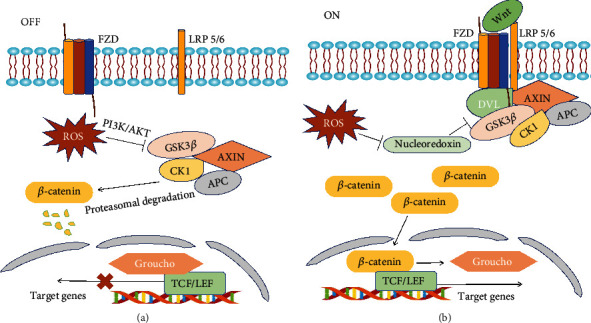
The transduction mechanism of the OS-mediated Wnt/*β*-catenin signaling pathway in OC. (a) In the absence of the Wnt ligand, the level of intracellular *β*-catenin is controlled by a “destruction complex” formed by glycogen synthase kinase 3*β* (GSK3*β*), casein kinase 1 (CK1), adenomatous polyposis coli (APC), and AXIN (a cytoplasmic protein regulating G-protein signaling) [138]. The destruction complex ubiquitinates and degrades *β*-catenin, thus preventing it from entering the nucleus to bind to the TCF/LEF complex and activate its target genes [132, 137]. ROS phosphorylate and inactivate GSK3*β* by activating the PI3K/AKT pathway and thus inhibiting GSK3*β*'s ability to degrade *β*-catenin [138, 139]. (b) In the presence of the Wnt ligand, the ligand binds to the cell surface receptor encompassing frizzled (FZD) and low-density lipoprotein receptor-related protein 5/6 (LRP5/6), leading to their conformational changes [137]. Disheveled (DVL) is then recruited and phosphorylated by FZD. Phosphorylated DVL in turn recruits AXIN, which inactivates the destruction complex and promotes the accumulation of *β*-catenin in the cytosol [138]. Subsequently, *β*-catenin is translocated into the nucleus where it displaces Groucho and binds to TCF/LEF members. Together with coactivators, the transcription of downstream target genes is initiated [132]. ROS can upregulate the Wnt pathway by oxidizing and inactivating nucleoredoxin, which inhibits DVL [136].

Another important signaling pathway in OC is Wnt/*β*-catenin, which plays a role in metastasis and therapy resistance [130–133]. Recent studies have also shown that it contributes to immune evasion of OC [134, 135]. NOX1-derived ROS have been reported to stimulate the Wnt/*β*-catenin pathway by oxidizing and inactivating nucleoredoxin (a redox-sensitive regulatory protein that negatively regulates the Wnt pathway by interacting with the Disheveled protein), thus promoting tumor cell proliferation [136] (Figure 5). As shown in Figure 5, in the absence of canonical Wnt ligands, the level of intracellular *β*-catenin is regulated by the polyprotein “destruction complex” [137]. CK1 and unphosphorylated GSK3*β* phosphorylate *β*-catenin and target it for degradation [133]. The PI3K/AKT pathway is activated by ROS and phosphorylates GSK3*β*, thus inhibiting its ability to phosphorylate and degrade *β*-catenin [138, 139] (Figure 5). Moreover, phosphorylated GSK3*β* is often found in OC [133]. A study showed that tankyrase (an oncogenic regulator of OC cell proliferation) promotes aerobic glycolysis of OC cells by stimulating the Wnt/*β*-catenin/Snail pathway [102]. Taken together, these results indicate that there is a bidirectional regulation between the Wnt/*β*-catenin pathway and the redox state of OC cells, and the interactions of these regulatory mechanisms are involved in the pathogenesis of OC.

Many reports on the invasive properties of OC have shown that the activation of epithelial-mesenchymal transition (EMT) is critical for the acquisition of a malignant phenotype in OC, especially in HGSC [140, 141]. OC cells undergoing EMT have stem cell-like properties that enable them to spread and metastasize [133]. The Wnt/*β*-catenin pathway is considered to be one of the main signaling pathways involved in EMT, and it plays a key role in the regulation and maintenance of OC stemness [142–144]. In addition, the Wnt/*β*-catenin signaling pathway is involved in the remodeling of the extracellular matrix in OC, which may be mediated by the activity of matrix metalloproteinase [132, 145].

A negative correlation has been demonstrated between Wnt activity and T cell signature [134, 146]. For example, Wnt inhibitors have been shown to significantly inhibit tumor progression and to increase the infiltration of CD8^+^ T cells in the TME in the OC model [146].

The Wnt pathway has been shown to be involved in drug resistance in OC, and inhibition of the Wnt pathway can increase the sensitivity of OC cells to chemotherapeutic agents [147]. In recent years, the Wnt pathway has also been shown to contribute to ameliorating adverse reactions caused by chemotherapy in OC. For example, in a mouse model of OC, the Wnt agonist BIO showed a significant therapeutic effect on cisplatin-induced acute kidney injury without affecting cisplatin's antitumor activity. One of the mechanisms was the activation of Wnt and its downstream pathway, which inhibits the production of excessive ROS in cells and thus reduces apoptosis in renal tubular cells [148].

#### 2.2.4. Notch Signaling Pathway

Notch and its intracellular domain (NICD) have been shown to be overexpressed in OC, and this is closely related to poorer prognosis in patients with OC [149–152]. OC cells in which Notch is activated show resistance to carboplatin, and it has been reported that methylseleninic acid can synergistically enhance the killing effect of carboplatin on OVCA429/NICD3 OC cells (which have a constitutively active form of Notch3) and that this can be promoted by ROS [153]. A correlation between Notch and NO/soluble guanylate cyclase (SGC) signaling has also been found. Low concentrations of NO can promote the progression of cancer, while many physiological functions of NO are mediated by SGC [103], and it has been confirmed that activation of the Notch pathway can enhance NO/SGC signaling in OC cells thereby promoting the proliferation and survival of OC cells [103]. In addition, a crosstalk has been shown between the Wnt/*β*-catenin and Notch pathways in OC. On the one hand, the *β*-catenin and Notch pathways synergistically promote proliferation and migration of OC cells, while on the other hand, inhibition of *β*-catenin increases the activity of the Notch system, thus showing the compensatory activities between the two pathways [154]. A method for detecting the activity of Notch has been developed that calculates the pathway activity score based upon the expression level of the conserved Notch target genes, and this has had a positive effect on clinical research and drug development for various diseases, including OC [155].

### 2.3. OS-Mediated Alterations of Transcription Factors in OC

In addition to signaling pathways, the development and progression of OC caused by OS is also closely related to several transcription factors. The following will focus on the regulation of OC by P53, NF-*κ*B, and HIF-1*α* under conditions of OS.

As a well-known tumor suppressor gene, p53 plays a major role in regulating cell proliferation, apoptosis, DNA repair, and genomic stability [156, 157], and about 96% of HGSC cases carry p53 mutations [158]. In contrast to wild-type p53, mutant p53 has been shown to stimulate the production of ROS by regulating redox-related signaling pathways and enzymes [159–161]. In addition, mutant p53 has been shown to activate glycolysis in tumor cells in order to maintain the Warburg effect, thus promoting cancer progression and tumor growth [159]. OS plays a role in the changes in p53 activity. OS has been shown to upregulate p53 in OC cells, thus inducing apoptosis and autophagy [162, 163]. In addition, high levels of OS can enhance the stability of mutant p53 [160, 164]. Interestingly, Padmanabhan et al. showed that apoptosis in OC cells was induced by OS and protein toxicity triggered by zinc oxide nanoparticles independently of the p53 mutation state [165].

NF-*κ*B is another important transcription factor involved in inflammation, immunity, apoptosis, and drug resistance [166, 167]. NF-*κ*B has been shown to contribute to the initiation of tumorigenesis and to play a crucial role in tumor cell proliferation and survival [168]. The p65 and p50 NF-*κ*B subunits have been shown to be highly expressed in OC patients and to be associated with poor prognosis [169]. NF-*κ*B can protect OC cells from OS by regulating the expression of antioxidant genes [170], and MnSOD (SOD2) seems to be the main target of NF-*κ*B [171]. Additionally, NF-*κ*B regulates the production of NO through iNOS, thus inducing angiogenesis and increasing resistance to apoptosis [172]. However, it has been shown that NF-*κ*B may also function as a potential tumor suppressor in some specific cases [168]. In contrast, ROS can promote the nuclear translocation of NF-*κ*B [173], and the inhibition of OS in OC cells has been shown to inactivate NF-*κ*B, thus inhibiting tumor progression [174]. Interestingly, Cys62, a key cysteine residue in the P50 domain, needs to be reduced in order to obtain effective NF-*κ*B DNA binding [173]. In addition, there is crosstalk between NF-*κ*B and Nrf2 under pathological conditions, and the two proteins inhibit each other [175].

Hypoxia-inducible factor 1 alpha (HIF-1*α*) is a key regulator of cellular response to hypoxia, which can be detected in many carcinomas, including OC. HIF-1*α* is closely related to tumor growth and angiogenesis [176, 177]. Under normal oxygen levels, HIF-1*α* is hydroxylated by prolyl hydroxylase (PHD), after which it binds to von Hippel-Lindau tumor suppressor protein and is subsequently ubiquitinated and degraded by proteasomes. However, under hypoxic conditions, the activity of PHD decreases, resulting in HIF-1*α* escaping from proteasome degradation [177, 178]. In addition to hydroxylation, SUMOylation and S-nitrosation are also related to the stability of HIF-1*α* [179]. Under hypoxic conditions, the levels of ROS in OC cells are increased paradoxically and this activates HIF-1*α* [178, 180]. HIF-1*α* has been shown to inhibit E-cadherin by upregulating the expression of its target genes, such as SNAIL [180], LOX [181], and AEG-1 [182], thus leading to EMT and promoting the invasion and metastasis of OC. Meanwhile, the reduced expression of HIF-1*α* suppresses the growth of OC cells [183, 184]. HIF-1*α* is also involved in the regulation of chemotherapy resistance, and it has been reported that HIF-1*α* can promote chemotherapy resistance by blocking the cell cycle in the G0/G1 phase [185]. Additionally, the tumor pharmacokinetic DCE-MRI perfusion parameters in patients with OC are negatively correlated with the expression level of HIF-1*α*, which can be used to screen the tumor characteristics of OC and help clinicians choose the best treatment options [186].

### 2.4. OS-Mediated Alterations of the TME in OC

The TME refers to the niche in which tumor cells interact with the host stroma, including different immune cells, fibroblasts, endothelial cells, and metabolites [187]. OC has a unique TME, and coevolution of cancer cells with their surroundings is an indispensable prerequisite for OC progression [188]. OS is involved in regulating OC progression by affecting components such as tumor-associated macrophages (TAMs), neutrophils, myeloid-derived suppressor cells (MDSCs), Treg cells, ascites, and lysophosphatidic acid (LPA) in the TME.

TAMs are a major inflammatory component of the tumor microenvironment and are associated with tumor growth and metastasis [189]. In TAMs, H_2_O_2_ triggers the expression of tumor necrosis factor-*α* (TNF-*α*) by activating the p38 and JNK pathways [190]. In turn, TNF induces ROS/RNS generation by controlling TNF signaling downstream of TNF receptors [191]. OS has been reported to drive TAMs to release different cytokines like TNF-*α*, interleukin1-*α* (IL1-*α*), IL-6, IL-10, and transforming growth factor *β* (TGF*β*) which results in the progression of OC [188, 192]. A study showed that OC cells can induce the production of itaconic acid (a metabolite resulting from tumor cell interactions with TAMs) in resident peritoneal macrophages, which contributed to the increase in oxidative phosphorylation and ROS and resulted in tumor growth [193].

Neutrophils, as the first line of defense against infection, are implicated in cancer-related inflammation [187]. Compared with the neutrophils of healthy women, the neutrophils isolated from OC patients show enhanced functional activity and higher ROS levels, which contribute to tumor progression and metastasis [194]. A study on dormancy models of OC and lung cancer showed that in response to stress hormones, the proinflammatory protein S100A8/A9 is released by neutrophils, and this leads to the accumulation of oxidized lipids in polymorphonuclear neutrophils by inducing the activation of myeloperoxidase. Moreover, oxidized lipids directly activate the proliferation of dormant tumor cells by upregulating the fibroblast growth factor receptor pathway [195].

MDSCs can inhibit immune responses in cancer patients and can lead to immune evasion [188] through the production and release of ROS and RNS [196] and are an important source of immunosuppression in OC [197]. The number of MDSCs is significantly increased in OC patients, especially monocytic MDSCs [198], and MDSCs can enhance the stemness of EOC cells by inducing the CSF2/p-STAT3 signaling pathway [199]. MDSCs can induce an increase in ROS [200], while ROS inhibitors can reverse MDSC-mediated T lymphocyte inhibition [201]. Another study showed that MDSCs generate NO through iNOS and that NO induces T cell apoptosis by inhibiting the Jak3/STAT5 signaling pathway [202]. In addition, tumor-infiltrating MDSCs have been shown to produce ONOO^–^, which nitrates tyrosine residues in the T cell receptor-CD8 complex, thus disrupting the binding of specific peptide-major histocompatibility complex dimers to CD8^+^ T cells [203].

Other cells in the TME are also involved in regulating tumor progression under conditions of OS. For example, Treg cells are implicated in tumor-associated immunosuppression and are vulnerable to OS, which has been shown to induce apoptosis in Treg cells. Interestingly, apoptotic Treg cells have been shown to enhance immunosuppression and to mediate the immune escape of tumor cells [204]. In addition, tumor-derived microvesicles contribute to restoring and improving the antigen processing capacity of clinical grade dendritic cells, which is related to the increase in ROS [205, 206].

Ascites is a key factor in the TME of OC and serves as a carrier to promote the spread of tumor cells to other pelvic and peritoneal organs [188], and OS plays an important role in this process [207]. Pakula et al. reported that malignant ascites generated by serous ovarian tumors triggers OS in human peritoneal mesothelial cells (HPMCs) by inducing cytochrome C oxidase and NADH dehydrogenase, which results in the senescence of HPMCs and thus promotes the adhesion, proliferation, and migration of OC cells [73]. In addition, malignant ascites can also increase the antioxidant capacity of OC cells [208]. GPx3 has been shown to be necessary for the survival of high-grade serous adenocarcinomas in ascites because it mediates the clearance of extracellular OS [209].

LPA, the second major group of lipids found in ascites [188], has been reported to be significantly increased in the ascites of OC patients and to promote the survival and proliferation of OC cells [210]. LPA stimulates the production of NOX-mediated ROS in OC cells, which is essential for the signal transduction of AKT, ERK, and NF-*κ*B, leading to the proliferation of OC cells [210, 211]. Blocking the LPA-dependent survival signaling pathway in OC cells has been shown to increase the production of ROS and to promote Taxol-induced apoptosis [212].

Hypoxia is a typical feature of the TME of malignant tumors and is attributed to the uncontrolled and rapid proliferation and irregular vascularization of the tumor [213]. Under hypoxic conditions, VEGF in malignant tumor cells is upregulated, which increases the metastatic ability of cancer cells [208].

## 3. Potential OS-Related Therapeutic Targets in OC

Based on the role of OS in OC, resolving the imbalance between oxidants and antioxidants is of great clinical importance in treating OC, and agents that modulate OS are regarded as an important choice for the prevention and treatment of OC. In recent years, many agents, including chemotherapeutic drugs, natural extracts and Chinese medicines, and nanoparticles, have received broad attention from the public. These agents play different roles in dealing with aberrant OS in OC. Most of these agents, such as berberine and methotrexate, work by promoting OS to induce oxidative damage to DNA and subsequent apoptosis in OC cells. Some of these agents such as *Ganoderma lucidum* and bisdemethoxycurcumin can activate antioxidant enzymes and reduce superoxide generation to decrease OS and inhibit the adhesion, invasion, and migration of OC cells.  Table 1 presents the three categories of therapeutic agents currently used for treating OC.

### 3.1. Chemotherapeutic Drugs

Chemotherapeutics can exhibit toxicity by inducing OS, inflammation, apoptosis, and abnormalities in neurotransmitter metabolism. ROS and RNS generated by anthracyclines and novel oxazolinoanthracyclines have drawn attention as novel signal mediators that are involved in the growth, differentiation, progression, and death of cancer cells [214]. Platinum coordination complexes, alkylating agents, camptothecins, and arsenic agents can induce high levels of ROS, while taxanes, vinca alkaloids, nucleotide analogues, and antimetabolites, including antifolates and nucleosides, generate lower levels of ROS [215]. Diosmetin has been shown to upregulate the levels of BAX while downregulating the expression of Bcl2, inhibiting Nrf2, and inducing the production of ROS [216]. Methotrexate has been shown to induce apoptosis in SKOV-3 cells via the ROS-mediated BAX/Bcl-2-Cyt-c release cascade [217]. PARP inhibitors have been shown to upregulate NADPH oxidases 1 and 4 and to have an antitumor effect by elevating OS in OC cells [218]. Cisplatin has been shown to downregulate HIF-1*α* in cisplatin-sensitive OC cells, and cisplatin plus-downregulated HIF-1*α* has been shown to induce apoptosis in cisplatin-resistant OC cells by inducing the overproduction of ROS [219]. In addition, the methylseleninic acid mentioned earlier, a promising future chemotherapeutic agent, has been shown to contribute to inhibiting OC progression by enhancing T cell-mediated tumor cell killing.

### 3.2. Natural Compounds and Chinese Medicines

Many extracts of natural compounds and Chinese medicines have been used in treating OC. Juglone (5-hydroxy-1, 4-napthoquinone) is isolated from various plants [220] and can increase ROS, resulting in ROS-dependent apoptosis by inducing cytochrome C and caspase-3, which are proapoptotic proteins involved in OS [221]. Moreover, juglone can upregulate *BAX* (a gene that promotes apoptosis) and downregulate *BCL2* (an apoptosis suppressor gene). Ailanthone, a natural compound extracted from the tree *Ailanthus altissima*, has been shown to decrease the proliferation and migration of cancer cells through a mechanism involving the posttranslational reduction of Nrf2 proteins, which in turn entails an increase in OS [222]. Olive leaf extract reduces OVCAR-3 cell viability by inducing cell cycle arrest, and it also induces apoptosis and increases the level of intracellular and mitochondrial ROS and decreases the activity of ROS scavenging enzymes [223]. Procyanidin, an extract from natural cocoa powder, increases ROS and activates caspase-3, thus leading to apoptosis [224]. Resveratrol (3,5,4′-trihydroxystilbene), from the roots of white hellebore and *Polygonum cuspidatum*, effectively induces OC stem cell death in a concentration-dependent manner, the mechanism of which might be through caspase-dependent apoptosis [225], the activation of caspase-9 and caspase-3 expression, and the downregulation of Notch expression [226]. The resveratrol derivative 3,3′,4,4′-tetrahydroxy-trans-stilbene also induces apoptosis and reduces proliferation via ROS-induced DNA damage [227]. Gedunin inhibits proliferation by upregulating cytochrome C and caspase-9/3, thus causing ROS-dependent apoptosis [228]. *Ganoderma lucidum* was demonstrated to induce the antioxidants SOD, CAT, NADPH, and GSTP1 via the Nrf2-mediated signaling pathway to provide chemoprotection against carcinogenicity [229]. *Antrodia salmonea* acts as a potent inducer of apoptosis in OC by upregulating the proapoptotic proteins caspase-9/3 and BAX, downregulating the antiapoptotic protein Bcl-2, and inactivating PI3K/AKT, all of which are mediated by ROS generation [129].

### 3.3. Nanoparticles

As emerging novel anticancer therapeutics, nanoparticles with good histocompatibility and targeting ability are becoming a hot research topic in the diagnosis and treatment of OC because of their small diameter and uniform distribution [230]. ZnO nanoparticles can induce severe oxidative and proteotoxic stress in OC cells through a dramatic decrease in intracellular glutathione levels [165], and ZnO nanoparticles with an average size of 20 nm were able to induce significant cytotoxicity in HOC cells by inducing increased levels of intracellular ROS [162]. SeChry and folate-targeted polyurea dendrimer generation four (SeChry@PURE_G4_-FA) nanoparticles can increase OS leading to GSH depletion and can inhibit the H_2_S-synthesizing enzyme cystathionine *β*-synthase, while upregulating the expression of the cystine/glutamate antiporter system Xc [231]. Celastrol is derived from the Chinese herb *Tripterygium wilfordii*, and nanoparticles loaded with celastrol have been designed to specifically target OC cells thus leading to increased intracellular ROS levels and apoptosis of tumor cells and thus achieving a therapeutic effect against OC [232]. These studies suggest that nanomaterials, especially nanomaterials combined with therapeutic agents, will play a crucial role in treating OC, and future research should focus on exploring the mechanisms through which these particles exert their effects on OC.

## 4. Concluding Remarks and Future Prospects

Accumulating evidence has shown the critical role of OS in the pathogenesis of OC via genetic changes and alterations to signaling pathways, transcription factors, and the TME. OS-mediated genetic alterations, such as oxidative damage to nuclear DNA and mtDNA, DNA hypomethylation, and abnormal expression of microRNA, can trigger OC. OS-mediated signaling pathways, such as Keap1-Nrf2-ARE, PI3K/AKT/mTOR, Wnt/*β*-catenin, and Notch, play important roles in regulating the progression of OC. Among these, activation of the Nrf2 pathway is considered to be a double-edged sword in OC because it maintains the stability of the normal ovarian cell environment and genome in order to prevent OS-induced carcinogenesis, while at the same time, it protects tumor cells from OS thereby enhancing the invasion and chemoresistance of OC. The PI3K/AKT/mTOR and Wnt/*β*-catenin pathways contribute to the proliferation, migration, and chemoresistance of OC, and they can be activated by OS. The OS-mediated Notch pathway is closely related to poor prognosis in patients with OC; however, the specific mechanism of action remains to be further studied. The transcription factors p53, NF-*κ*B, and HIF-1*α* are also ROS sensitive, and redox modification of these molecules can be instrumental in the initiation and progression of OC. Finally, OS is involved in regulating OC progression by affecting components such as TAMs, neutrophils, MDSCs, Treg cells, ascites, and LPA in the TME of OC.

We have also outlined three treatment strategies to target aberrant OS in OC, including chemotherapeutic drugs, natural compound extracts or Chinese medicine, and nanoparticles. Their mechanisms of action may be through affecting oxidative damage to DNA, by regulating signaling pathways such as Notch and Keap1-Nrf2-ARE and transcription factors such as HIF-1*α* and by affecting components of the TME. These agents play different roles in dealing with aberrant OS in OC. Most of them can promote OS to induce DNA oxidative damage and ROS-dependent apoptosis of OC cells, while some agents can activate antioxidant enzymes and reduce superoxide generation to decrease OS. All of them contribute to inhibiting the adhesion, invasion, and migration of OC cells. There is a multitarget effect between OC and OS, and future experiments in clinic are needed to validate the relationship between OS and OC.

## Figures and Tables

**Table 1:** Agents targeting OS in OC.

Category	Materials	Cell lines	Targets	Mechanism	Effects	Refs.
Chemotherapeutic drugs	Diosmetin	A2780 and SKOV3 cells	BAX↑, Bcl-2↓, Nrf2↓	ROS-mediated apoptosis	Inhibiting proliferation, migration, and invasion	[216]
	Methotrexate	SKOV3 cells	BAX↑, Bcl-2↓, cytochrome C↑	ROS-induced DNA damage	Inducing apoptosis	[217]
	PARP inhibitors	A2780 and HO8910 cells	NOX 1↑, NOX 4↑	ROS-dependent apoptosis	Inducing apoptosis	[218]

Natural compounds and Chinese medicines	Juglone	SKOV3 cells	Cytochrome C↑, caspase-3↑	ROS-dependent apoptosis	Inhibiting proliferation	[221]
	Ailanthone	A2780 and A2780/CP70 cells	Nrf2↓	OS↑	Reducing proliferation and migration	[222]
	Olive leaf extract	OVCAR-3 and OVCAR-8 cells	Caspase 9↑, SOD↓, CAT↓	ROS-dependent apoptosis	Suppressing proliferation, cell cycle progression, and aggregation	[223]
	Procyanidin	OAW42 and OVCAR3 cells	MMP↓, caspase-3↑	ROS/caspase-3-mediated apoptosis	Inducing cell death and inhibiting invasion	[224]
	Gossypol	SKOV3 cells	GSH↓, FAD↓	ROS apoptosis	Increasing apoptosis	[237]
	Resveratrol	A2780 and SKOV-3 cells	Caspase-9 and caspase-3↑, Notch↓	ROS-dependent apoptosis	Inducing cell death	[226]
	Resveratrol derivative	A2780, SKOV-3, and OVCAR-3 cells	SOD↓, CAT↓, 8-OHdG↑	ROS-induced DNA damage	Inducing apoptosis, reducing proliferation, and activating senescence	[227]
	Gedunin	PA-1 and OVCAR-3 cells	Cytochrome C↑, caspase-9 and caspase-3↑	ROS-dependent apoptosis	Inhibiting proliferation	[228]
	*Ganoderma lucidum*	OVCAR-3 cells	SOD↑, CAT↑, GSTP1↑, H_2_O_2_↓, Nrf2-Keap1 signaling↑	OS↓	Inhibiting proliferation	[229]
	Berberine	A2780, HEY, SKOV-3, HO8910, HO8910PM, and OVCAR3 cells	8-OHdG↑	ROS-induced DNA damage	Increasing apoptosis and reducing cell growth	[238]
	Cucurbitacin I	SKOV-3 cells	Caspase-3↑, BAX↑, Bcl-2↓, Nrf2-Keap1 signaling↓	ROS-mediated apoptosis	Inducing cell death	[239]
	Bisdemethoxycurcumin	SKOV-3 cells	Superoxide↓	OS↓	Inhibiting adhesion, invasion, and migration	[174]
	*Antrodia salmonea*	SKOV-3 and A2780 cells	Caspase-9, caspase-3↑, BAX↑, Bcl-2↓	ROS-dependent apoptosis	Inducing cell death	[129]
	*Vernonia calvoana*	OVCAR-3	GSH↓, CAT↓	ROS-induced DNA damage	Suppressing cell proliferation	[240]
Nanoparticles	ZnO nanoparticles	SKOV-3, 3T3-L1 cells, TYKNu, ALST, OVCAR3, and OVCA420	Caspase-3↑, GSH↓	ROS-mediated apoptosis	Inducing cell death	[165]
	SeChry@PUREG4-FA	OVCAR3 HTB-161, OVCAR8 CVCL-1629, and ES2 CRL-1978™	GSH↓, CBS↓	ROS-mediated apoptosis	Increasing cell death; reducing toxicity of nonmalignant cells	[231]
	Celastrol-loaded nanoparticles	SKOV-3 cells	GSH↓	ROS-dependent apoptosis	Inhibiting cell migration and invasion	[232]
	Flavonoids	A2780, OVCAR-3, and SKOV-3	Caspase-3↑	ROS/caspase-3-mediated apoptosis	Inducting apoptosis and reducing invasion	[241]
	CYT-Rx20	MDAH 2774, PA-1, and SKOV3 cells	Caspase-9, caspase-3↑	ROS-dependent apoptosis	Reducing cell viability and inducing cell death	[242]
	Sideroxylin	ES2 and OV90 cells	MAPK and PI3-K pathway transduction↑	ROS-mediated apoptosis	Suppressing cell proliferation and increasing apoptosis	[243]
	Morusin	A2780, SKOV-3, and HO-8910	Mitochondrial Ca2^+^↑	ROS-dependent apoptosis	Inhibiting cell proliferation and survival	[244]

8-OHdG: 8-hydroxy-2′-deoxyguanosin; CAT: catalase; CBS: cystathionine *β*-synthase; FAD: flavin adenine dinucleotide; GSH: glutathione; GSTP1: glutathione S-transferase P1; H_2_O_2_: hydrogen peroxide; Keap1: Kelch-like ECH-associated protein 1; MAPK: mitogen-activated protein kinase; MMP: matrix metalloproteinase; NOX: nicotinamide adenine dinucleotide oxidase; Nrf2: nuclear factor E2-related factor 2; OS: oxidative stress; PI3K: phosphatidylinositol 3-kinase; ROS: reactive oxygen species; SOD: superoxide dismutase.

## Abbreviations

8-OHdG: 8-Hydroxy-2′-deoxyguanosin
AKT: Protein kinase B
APC: Adenomatous polyposis coli
ATF: Artificial transcription factor
CAT: Catalase
CBS: Cystathionine *β*-synthase
CK1: Casein kinase 1
Cyt C: Cytochrome C
DVL: Disheveled
EGF: Epidermal growth factor
EMT: Epithelial-mesenchymal transition
EOC: Epithelial ovarian cancer
FAD: Flavin adenine dinucleotide
FZD: Frizzled
G6PD: Glucose-6-phosphate dehydrogenase
GPX: Glutathione peroxidase
GSH: Glutathione
GSK3*β*: Glycogen synthase kinase 3*β*
GSR: Glutathione reductase
GST: Glutathione S-transferases
H_2_O_2_: Hydrogen peroxide
HIF-1*α*: Hypoxia-inducible factor 1 alpha
HGSC: High-grade serous carcinoma
HPMCs: Human peritoneal mesothelial cells
HRR: Homologous recombination repair
IL: Interleukin
iNOS: Inducible nitric oxide synthase
Keap1: Kelch-like ECH-associated protein 1
LPA: Lysophosphatidic acid
Maf: Musculoaponeurotic fibrosarcoma
MAPK: Mitogen-activated protein kinase
MDSCs: Myeloid-derived suppressor cells
MIEF2: Mitochondrial elongation factor 2
miRNA: MicroRNA
mtDNA: Mitochondrial DNA
mTOR: Mammalian target of rapamycin
mTORC1: MTOR complex 1
NADPH: Nicotinamide adenine dinucleotide
NICD: The intracellular domain of Notch
NO: Nitric oxide
NOX: Nicotinamide adenine dinucleotide oxidase
Nrf2: Nuclear factor E2-related factor 2
OC: Ovarian cancer
^1^O_2_: Singlet oxygen
O_2_·^–^: Superoxide anion
OH: Hydroxyl radical
ONOO^−^: Peroxynitrite anion
OS: Oxidative stress
PGC1*α*: Peroxisome proliferator-activated receptor-*γ* coactivator 1*α*
PHD: Prolyl hydroxylase
PI3K: Phosphatidylinositol 3-kinase
PIP3: Phosphatidylinositol trisphosphate
PIP2: Phosphorylates phosphatidylinositol bisphosphate
PPP: Pentose phosphate pathway
PRX: Peroxiredoxin
PTEN: Phosphatase and tensin homolog
PTP: Protein tyrosine phosphatase
ROS: Reactive oxygen
RNS: Reactive nitrogen species
RTK: Receptor tyrosine kinase
SeChry@PUREG4-FA: SeChry and folate-targeted polyurea dendrimer generation four
SGC: Soluble guanylate cyclase
SOD: Superoxide dismutase
SOD2: MnSOD
TAMs: Tumor-associated macrophages
Tf: Transferrin
TGF*β*: Transforming growth factor *β*
TME: Tumor microenvironment
TNF: Tumor necrosis factor
TRX: Thioredoxin
VEGF: Vascular endothelial growth factor
VHL: von Hippel-Lindau tumor suppressor protein
XO: Xanthine oxidase.

## References

[1] Bray F., Ferlay J., Soerjomataram I., Siegel R. L., Torre L. A., Jemal A. (2018). Global Cancer Statistics 2018: GLOBOCAN estimates of incidence and mortality worldwide for 36 cancers in 185 countries. *CA: A Cancer Journal for Clinicians*.

[2] Webb P. M., Jordan S. J. (2017). Epidemiology of epithelial ovarian cancer. *Best Practice & Research. Clinical Obstetrics & Gynaecology*.

[3] Kurman R. J., Shih I.-M. (2016). The Dualistic Model of Ovarian Carcinogenesis: Revisited, Revised, and Expanded. *The American Journal of Pathology*.

[4] Ducie J., Dao F., Considine M. (2017). Molecular analysis of high-grade serous ovarian carcinoma with and without associated serous tubal intra-epithelial carcinoma. *Nature Communications*.

[5] Bowtell D. D., Böhm S., Ahmed A. A. (2015). Rethinking ovarian cancer Ii: reducing mortality from high-grade serous ovarian cancer. *Nature Reviews Cancer*.

[6] Soong T. R., Dinulescu D. M., Xian W., Crum C. P. (2018). Frontiers in the Pathology and Pathogenesis of Ovarian Cancer: Cancer Precursors and "Precursor Escape". *Hematology/Oncology Clinics of North America*.

[7] Lheureux S., Gourley C., Vergote I., Oza A. M. (2019). Epithelial ovarian cancer. *The Lancet*.

[8] Matulonis U. A., Sood A. K., Fallowfield L., Howitt B. E., Sehouli J., Karlan B. Y. (2016). Ovarian cancer. *Nature Reviews. Disease Primers*.

[9] Ham J., Lim W., Kim K. (2019). Gentisyl alcohol inhibits proliferation and induces apoptosis via mitochondrial dysfunction and regulation of Mapk and Pi3k/Akt pathways in epithelial ovarian cancer cells. *Marine Drugs*.

[10] Ďuračková Z. (2010). Some Current Insights into Oxidative Stress. *Physiological Research*.

[11] Reuter S., Gupta S. C., Chaturvedi M. M., Aggarwal B. B. (2010). Oxidative stress, inflammation, and cancer: how are they linked?. *Free Radical Biology & Medicine*.

[12] Pisoschi A. M., Pop A. (2015). The role of antioxidants in the chemistry of oxidative stress: a review. *European Journal of Medicinal Chemistry*.

[13] Navarro-Yepes J., Zavala-Flores L., Anandhan A. (2014). Antioxidant Gene Therapy against Neuronal Cell Death. *Pharmacology & Therapeutics*.

[14] Sharaf A., de Michele, Sharma A., Fakhari S., Oborník (2019). Transcriptomic analysis reveals the roles of detoxification systems in response to mercury in Chromera Velia. *Biomolecules*.

[15] Dröge W. (2002). Free radicals in the physiological control of cell function. *Physiological Reviews*.

[16] Saed G. M., Diamond M. P., Fletcher N. M. (2017). Updates of the role of oxidative stress in the pathogenesis of ovarian cancer. *Gynecologic Oncology*.

[17] Shankar K., Mehendale H. M. (2014). Oxidative stress. *Encyclopedia of Toxicology*.

[18] Basak D., Uddin M. N., Hancock J. (2020). The role of oxidative stress and its counteractive utility in colorectal cancer (Crc). *Cancers*.

[19] Oyewole A. O., Birch-Machin M. A. (2015). Mitochondria-targeted antioxidants. *The FASEB Journal*.

[20] Vilchez A., Acevedo F., Cea M., Seeger M., Navia R. (2020). Applications of electrospun nanofibers with antioxidant properties: a review. *Nanomaterials*.

[21] Ismail T., Kim Y., Lee H., Lee D. S., Lee H. S. (2019). Interplay between mitochondrial peroxiredoxins and Ros in cancer development and progression. *International Journal of Molecular Sciences*.

[22] Finkel T. (2011). Signal transduction by reactive oxygen species. *Journal of Cell Biology*.

[23] Veskoukis A. S., Tsatsakis A. M., Kouretas D. (2012). Dietary oxidative stress and antioxidant defense with an emphasis on plant extract administration. *Cell Stress & Chaperones*.

[24] Benhar M. (2018). Roles of mammalian glutathione peroxidase and thioredoxin reductase enzymes in the cellular response to nitrosative stress. *Free Radical Biology & Medicine*.

[25] Kirtonia A., Sethi G., Garg M. (2020). The multifaceted role of reactive oxygen species in tumorigenesis. *Cellular and Molecular Life Sciences*.

[26] Di Meo S., Venditti P. (2020). Evolution of the knowledge of free radicals and other oxidants. *Oxidative Medicine and Cellular Longevity*.

[27] Bakadia B. M., Boni B. O. O., Ahmed A. A. Q., Yang G. (2021). The impact of oxidative stress damage induced by the environmental stressors on Covid-19. *Life Sciences*.

[28] Pizzino G., Irrera N., Cucinotta M. (2017). Oxidative stress: harms and benefits for human health. *Oxidative Medicine and Cellular Longevity*.

[29] Gautam V., Kohli S. K., Arora S. (2018). Antioxidant and antimutagenic activities of different fractions from the leaves of Rhododendron arboreum Sm. and their Gc-Ms profiling. *Molecules*.

[30] Wang Q., Guo W., Hao B. (2016). Mechanistic study of TRPM2-Ca^2+^-CAMK2-BECN1 signaling in oxidative stress-induced autophagy inhibition. *Autophagy*.

[31] Hayes J. D., Dinkova-Kostova A. T., Tew K. D. (2020). Oxidative stress in cancer. *Cancer Cell*.

[32] Lee J., Giordano S., Zhang J. (2012). Autophagy, mitochondria and oxidative stress: cross-talk and redox signalling. *Biochemical Journal*.

[33] Sosa V., Moliné T., Somoza R., Paciucci R., Kondoh H., Lleonart M. E. (2013). Oxidative stress and cancer: an overview. *Ageing Research Reviews*.

[34] Kumari S., Badana A. K., G M. M., G S., Malla R. R. (2018). Reactive oxygen species: a key constituent in cancer survival. *Biomarker Insights*.

[35] Caneba C. A., Yang L., Baddour J. (2014). Nitric oxide is a positive regulator of the Warburg effect in ovarian cancer cells. *Cell Death & Disease*.

[36] Galadari S., Rahman A., Pallichankandy S., Thayyullathil F. (2017). Reactive oxygen species and cancer paradox: to promote or to suppress?. *Free Radical Biology & Medicine*.

[37] Dodson M., Castro-Portuguez R., Zhang D. D. (2019). Nrf2 plays a critical role in mitigating lipid peroxidation and ferroptosis. *Redox Biology*.

[38] Redza-Dutordoir M., Averill-Bates D. A. (2016). Activation of apoptosis signalling pathways by reactive oxygen species. *Biochimica et Biophysica Acta (BBA) - Molecular Cell Research*.

[39] Jiang Z., Fletcher N. M., Ali-Fehmi R. (2011). Modulation of redox signaling promotes apoptosis in epithelial ovarian cancer cells. *Gynecologic Oncology*.

[40] Graham K. A., Kulawiec M., Owens K. M. (2010). NADPH oxidase 4 is an oncoprotein localized to mitochondria. *Cancer Biology & Therapy*.

[41] Nomelini R. S., Ribeiro L. C. . A., Tavares-Murta B. M., Adad S. J., Murta E. F. (2008). Production of nitric oxide and expression of inducible nitric oxide synthase in ovarian cystic tumors. *Mediators of Inflammation*.

[42] Hu Y., Rosen D. G., Zhou Y. (2005). Mitochondrial Manganese-Superoxide Dismutase Expression in Ovarian Cancer:. *Journal of Biological Chemistry*.

[43] Hemachandra L. P., Shin D. H., Dier U. (2015). Mitochondrial superoxide dismutase has a protumorigenic role in ovarian clear cell carcinoma. *Cancer Research*.

[44] Chatterjee A., Gupta S. (2018). The multifaceted role of glutathione S-transferases in cancer. *Cancer Letters*.

[45] Liberti M. V., Locasale J. W. (2016). The Warburg effect: how does it benefit cancer cells?. *Trends in Biochemical Sciences*.

[46] Pritchard K. I., Julian J. A., Holloway C. M. (2012). Prospective study of 2-[18F]Fluorodeoxyglucose positron emission tomography in the assessment of regional nodal spread of disease in patients with breast cancer: an Ontario Clinical Oncology Group study. *Journal of Clinical Oncology*.

[47] Amano T., Murakami A., Murakami T., Chano T. (2021). Antioxidants and therapeutic targets in ovarian clear cell carcinoma. *Antioxidants*.

[48] Weinberg F., Chandel N. S. (2009). Mitochondrial metabolism and cancer. *Annals of the New York Academy of Sciences*.

[49] Kuehne A., Emmert H., Soehle J. (2015). Acute activation of oxidative pentose phosphate pathway as first-line response to oxidative stress in human skin cells. *Molecular Cell*.

[50] Ying W. (2008). NAD+/NADH and NADP+/NADPH in cellular functions and cell death: regulation and biological consequences. *Antioxidants & Redox Signaling*.

[51] Purohit V., Simeone D. M., Lyssiotis C. A. (2019). Metabolic regulation of redox balance in cancer. *Cancers*.

[52] Ding Y., Gong C., Huang D. (2018). Synthetic lethality between Her2 and transaldolase in intrinsically resistant Her2-positive breast cancers. *Nature Communications*.

[53] de Raedt T., Walton Z., Yecies J. L. (2011). Exploiting cancer cell vulnerabilities to develop a combination therapy for Ras-driven tumors. *Cancer Cell*.

[54] Blackburn A. C., Matthaei K. I., Lim C. (2006). Deficiency of glutathione transferase zeta causes oxidative stress and activation of antioxidant response pathways. *Molecular Pharmacology*.

[55] Zheng L., Cardaci S., Jerby L. (2015). Fumarate induces redox-dependent senescence by modifying glutathione metabolism. *Nature Communications*.

[56] Chen Y., Singh S., Matsumoto A. (2016). Chronic glutathione depletion confers protection against alcohol-induced steatosis: implication for redox activation of Amp-activated protein kinase pathway. *Scientific Reports*.

[57] Marinho H. S., Real C., Cyrne L., Soares H., Antunes F. (2014). Hydrogen peroxide sensing, signaling and regulation of transcription factors. *Redox Biology*.

[58] Faes S., Dormond O. (2015). Pi3k and Akt: unfaithful partners in cancer. *International Journal of Molecular Sciences*.

[59] Ray P. D., Huang B. W., Tsuji Y. (2012). Reactive oxygen species (ROS) homeostasis and redox regulation in cellular signaling. *Cellular Signalling*.

[60] Kleih M., Bopple K., Dong M. (2019). Direct impact of cisplatin on mitochondria induces ROS production that dictates cell fate of ovarian cancer cells. *Cell Death & Disease*.

[61] Mosca L., Ilari A., Fazi F., Assaraf Y. G., Colotti G. (2021). Taxanes in cancer treatment: activity, chemoresistance and its overcoming. *Drug Resistance Updates*.

[62] Yamada Y., Shigetomi H., Onogi A. (2011). Redox-active iron-induced oxidative stress in the pathogenesis of clear cell carcinoma of the ovary. *International Journal of Gynecological Cancer*.

[63] Iwabuchi T., Yoshimoto C., Shigetomi H., Kobayashi H. (2015). Oxidative stress and antioxidant defense in endometriosis and its malignant transformation. *Oxidative Medicine and Cellular Longevity*.

[64] Kobayashi H., Ogawa K., Kawahara N. (2017). Sequential molecular changes and dynamic oxidative stress in high-grade serous ovarian carcinogenesis. *Free Radical Research*.

[65] Marí-Alexandre, Pellín Carcelén, Agababyan C. (2019). Interplay between microRNAs and oxidative stress in ovarian conditions with a focus on ovarian cancer and endometriosis. *International Journal of Molecular Sciences*.

[66] Calaf G. M., Urzua U., Termini L., Aguayo F. (2018). Oxidative stress in female cancers. *Oncotarget*.

[67] Klaunig J. E., Kamendulis L. M. (2004). Therole Ofoxidativestress Incarcinogenesis. *Annual Review of Pharmacology and Toxicology*.

[68] Yang Y., Karakhanova S., Werner J., Bazhin A. V. (2013). Reactive oxygen species in cancer biology and anticancer therapy. *Current Medicinal Chemistry*.

[69] Shigeta S., Toyoshima M., Kitatani K., Ishibashi M., Usui T., Yaegashi N. (2016). Transferrin facilitates the formation of DNA double-strand breaks via transferrin receptor 1: the possible involvement of transferrin in carcinogenesis of high-grade serous ovarian cancer. *Oncogene*.

[70] Ye W., Zhang Y., Hu W., Wang L., Zhang Y., Wang P. (2020). A sensitive FRET biosensor based on carbon dots-modified nanoporous membrane for 8-hydroxy-2′-Deoxyguanosine (8-OHdG) detection with Au@Zif-8 nanoparticles as signal quenchers. *Nanomaterials*.

[71] Valavanidis A., Vlachogianni T., Fiotakis C. (2009). 8-hydroxy-2′-deoxyguanosine (8-OHdG): a critical biomarker of oxidative stress and carcinogenesis. *Journal of Environmental Science and Health, Part C*.

[72] Hwang E. S., Bowen P. E. (2007). DNA damage, a biomarker of carcinogenesis: its measurement and modulation by diet and environment. *Critical Reviews in Food Science and Nutrition*.

[73] Pakuła M., Mikuła-Pietrasik J., Stryczyński Ł. (2018). Mitochondria-related oxidative stress contributes to ovarian cancer-promoting activity of mesothelial cells subjected to malignant ascites. *The International Journal of Biochemistry & Cell Biology*.

[74] van Loon B., Markkanen E., Hubscher U. (2010). Oxygen as a friend and enemy: how to combat the mutational potential of 8-oxo-guanine. *DNA Repair*.

[75] Xu L., Wu T., Lu S. (2020). Mitochondrial superoxide contributes to oxidative stress exacerbated by DNA damage response in RAD51-depleted ovarian cancer cells. *Redox Biology*.

[76] Ghosh A., Bhattacharjee S., Chowdhuri S. P. (2019). SCAN1-TDP1 trapping on mitochondrial DNA promotes mitochondrial dysfunction and mitophagy. *Science Advances*.

[77] Abedi S., Yung G., Atilano S. R. (2020). Differential effects of cisplatin on cybrid cells with varying mitochondrial DNA haplogroups. *PeerJ*.

[78] van Gisbergen M. W., Voets A. M., Starmans M. H. (2015). How do changes in the mtDNA and mitochondrial dysfunction influence cancer and cancer therapy? Challenges, opportunities and models. *Mutation Research, Reviews in Mutation Research*.

[79] Liu V. W., Shi H. H., Cheung A. N. (2001). High incidence of somatic mitochondrial DNA mutations in human ovarian carcinomas. *Cancer Research*.

[80] Wallace D. C. (2012). Mitochondria and cancer. *Nature Reviews Cancer*.

[81] Van Trappen P. O., Cullup T., Troke R. (2007). Somatic mitochondrial DNA mutations in primary and metastatic ovarian cancer. *Gynecologic Oncology*.

[82] Shay J. W., Werbin H. (1987). Are mitochondrial DNA mutations involved in the carcinogenic process?. *Mutation Research/Reviews in Genetic Toxicology*.

[83] Saki M., Prakash A. (2017). DNA damage related crosstalk between the nucleus and mitochondria. *Free Radical Biology & Medicine*.

[84] Fang E. F., Scheibye-Knudsen M., Chua K. F., Mattson M. P., Croteau D. L., Bohr V. A. (2016). Nuclear DNA damage signalling to mitochondria in ageing. *Nature Reviews Molecular Cell Biology*.

[85] Yang Y., Karakhanova S., Hartwig W. (2016). Mitochondria and mitochondrial ROS in cancer: novel targets for anticancer therapy. *Journal of Cellular Physiology*.

[86] Ni J., Wang Y., Cheng X. (2020). Pathogenic heteroplasmic somatic mitochondrial DNA mutation confers platinum-resistance and recurrence of high-grade serous ovarian Cancer. *Cancer Management and Research*.

[87] Guerra F., Perrone A. M., Kurelac I. (2012). Mitochondrial DNA mutation in serous ovarian cancer: implications for mitochondria-coded genes in chemoresistance. *Journal of Clinical Oncology*.

[88] Ehrlich M. (2009). DNA hypomethylation in cancer cells. *Epigenomics*.

[89] Luo C., Hajkova P., Ecker J. R. (2018). Dynamic DNA methylation: in the right place at the right time. *Science*.

[90] Wu Q., Ni X. (2015). ROS-mediated DNA methylation pattern alterations in carcinogenesis. *Current Drug Targets*.

[91] Maltseva D. V., Baykov A. A., Jeltsch A., Gromova E. S. (2009). Impact of 7,8-dihydro-8-oxoguanine on methylation of the CpG site by Dnmt3a. *Biochemistry*.

[92] Koczor C. A., Ludlow I., Fields E. (2016). Mitochondrial polymerase gamma dysfunction and aging cause cardiac nuclear DNA methylation changes. *Physiological Genomics*.

[93] Hiura H., Okae H., Kobayash H. (2012). High-throughput detection of aberrant imprint methylation in the ovarian cancer by the bisulphite PCR-Luminex method. *BMC Medical Genomics*.

[94] Qin L., Li T., Liu Y. (2017). High Slc4a11 expression is an independent predictor for poor overall survival in grade 3/4 serous ovarian cancer. *PLoS One*.

[95] Batista L., Gruosso T., Mechta-Grigoriou F. (2013). Ovarian cancer emerging subtypes: role of oxidative stress and fibrosis in tumour development and response to treatment. *The International Journal of Biochemistry & Cell Biology*.

[96] Cuzziol C. I., Castanhole-Nunes M. M. U., Pavarino E. C., Goloni-Bertollo E. M. (2020). MicroRNAs as regulators of *VEGFA* and *NFE2L2* in cancer. *Gene*.

[97] Acuna S. M., Floeter-Winter L. M., Muxel S. M. (2020). MicroRNAs: biological regulators in pathogen-host interactions. *Cell*.

[98] Brozovic A., Duran G. E., Wang Y. C., Francisco E. B., Sikic B. I. (2015). The mir-200 family differentially regulates sensitivity to paclitaxel and carboplatin in human ovarian carcinoma OVCAR-3 and MES-OV cells. *Molecular Oncology*.

[99] Mateescu B., Batista L., Cardon M. (2011). mir-141 and mir-200a act on ovarian tumorigenesis by controlling oxidative stress response. *Nature Medicine*.

[100] Zhao S., Cheng L., Shi Y., Li J., Yun Q., Yang H. (2021). MIEF2 reprograms lipid metabolism to drive progression of ovarian cancer through ROS/AKT/mTOR signaling pathway. *Cell Death & Disease*.

[101] Deng X., Lin N., Fu J. (2020). The Nrf2/PGC1*α* Pathway Regulates Antioxidant and Proteasomal Activity to Alter Cisplatin Sensitivity in Ovarian Cancer. *Oxidative Medicine and Cellular Longevity*.

[102] Yang H. Y., Shen J. X., Wang Y., Liu Y., Shen D. Y., Quan S. (2019). Tankyrase promotes aerobic glycolysis and proliferation of ovarian cancer through activation of Wnt/beta-catenin signaling. *BioMed Research International*.

[103] El-Sehemy A., Chang A. C., Azad A. K. (2013). Notch activation augments nitric oxide/soluble guanylyl cyclase signaling in immortalized ovarian surface epithelial cells and ovarian cancer cells. *Cellular Signalling*.

[104] Wu X., Han L. Y., Zhang X. X., Wang L. (2018). The study of Nrf2 signaling pathway in ovarian cancer. *Critical Reviews in Eukaryotic Gene Expression*.

[105] Niture S. K., Kaspar J. W., Shen J., Jaiswal A. K. (2010). Nrf2 signaling and cell survival. *Toxicology and Applied Pharmacology*.

[106] Villeneuve N. F., Lau A., Zhang D. D. (2010). Regulation of the Nrf2-Keap1 antioxidant response by the ubiquitin proteasome system: an insight into cullin-ring ubiquitin ligases. *Antioxidants & Redox Signaling*.

[107] Li D., Hong X., Zhao F., Ci X., Zhang S. (2021). Targeting Nrf2 may reverse the drug resistance in ovarian cancer. *Cancer Cell International*.

[108] Ganan-Gomez I., Wei Y., Yang H., Boyano-Adanez M. C., Garcia-Manero G. (2013). Oncogenic functions of the transcription factor Nrf2. *Free Radical Biology & Medicine*.

[109] Khalil H. S., Goltsov A., Langdon S. P., Harrison D. J., Bown J., Deeni Y. (2015). Quantitative analysis of Nrf2 pathway reveals key elements of the regulatory circuits underlying antioxidant response and proliferation of ovarian cancer cells. *Journal of Biotechnology*.

[110] Sirota R., Gibson D., Kohen R. (2015). The role of the catecholic and the electrophilic moieties of caffeic acid in Nrf2/Keap1 pathway activation in ovarian carcinoma cell lines. *Redox Biology*.

[111] Lang F., Qu J., Yin H. (2018). Apoptotic cell death induced by Z-ligustilidein human ovarian cancer cells and role of Nrf2. *Food and Chemical Toxicology*.

[112] van der Wijst M. G., Huisman C., Mposhi A., Roelfes G., Rots M. G. (2015). Targeting Nrf2 in healthy and malignant ovarian epithelial cells: protection versus promotion. *Molecular Oncology*.

[113] Xia M. H., Yan X. Y., Zhou L. (2020). P62 suppressed Vk3-induced oxidative damage through Keap1/Nrf2 pathway in human ovarian cancer cells. *Journal of Cancer*.

[114] Liu N., Lin X., Huang C. (2020). Activation of the reverse transsulfuration pathway through NRF2/CBS confers erastin-induced ferroptosis resistance. *British Journal of Cancer*.

[115] Xia M., Yu H., Gu S. (2014). P62/Sqstm1 is involved in cisplatin resistance in human ovarian cancer cells via the Keap1-Nrf2-Are system. *International Journal of Oncology*.

[116] Konstantinopoulos P. A., Spentzos D., Fountzilas E. (2011). Keap1 mutations and Nrf2 pathway activation in epithelial ovarian cancer. *Cancer Research*.

[117] Wu J., Zhang L., Li H., Wu S., Liu Z. (2019). Nrf2 induced cisplatin resistance in ovarian cancer by promoting Cd99 expression. *Biochemical and Biophysical Research Communications*.

[118] Cho H.-y., Kim K., Kim Y.-B., Kim H., No J. H. (2017). Expression patterns of Nrf2 and Keap1 in ovarian cancer cells and their prognostic role in disease recurrence and patient survival. *International Journal of Gynecological Cancer*.

[119] Aziz A. U. R., Farid S., Qin K., Wang H., Liu B. (2018). PIM kinases and their relevance to the PI3K/AKT/mTOR pathway in the regulation of ovarian cancer. *Biomolecules*.

[120] Dobbin Z. C., Landen C. N. (2013). The importance of the PI3K/AKT/mTOR pathway in the progression of ovarian cancer. *International Journal of Molecular Sciences*.

[121] Ediriweera M. K., Tennekoon K. H., Samarakoon S. R. (2019). Role of the PI3K/AKT/mTOR signaling pathway in ovarian cancer: biological and therapeutic significance. *Seminars in Cancer Biology*.

[122] Mabuchi S., Kuroda H., Takahashi R., Sasano T. (2015). The PI3K/AKT/mTOR pathway as a therapeutic target in ovarian cancer. *Gynecologic Oncology*.

[123] Sakamoto K., Iwasaki K., Sugiyama H., Tsuji Y. (2009). Role of the tumor suppressor PTEN in antioxidant responsive element-mediated transcription and associated histone modifications. *Molecular Biology of the Cell*.

[124] van der Reest J., Lilla S., Zheng L., Zanivan S., Gottlieb E. (2018). Proteome-wide analysis of cysteine oxidation reveals metabolic sensitivity to redox stress. *Nature Communications*.

[125] Wu W. S. (2007). The signaling mechanism of ROS in tumor progression. *Cancer and Metastasis Reviews*.

[126] Lee Y. K., Park N. H. (2009). Prognostic value and clinicopathological significance of *p53* and *PTEN* in epithelial ovarian cancers. *Gynecologic Oncology*.

[127] Liu L. Z., Hu X. W., Xia C. (2006). Reactive oxygen species regulate epidermal growth factor-induced vascular endothelial growth factor and hypoxia-inducible factor-1*α* expression through activation of AKT and P70S6K1 in human ovarian cancer cells. *Free Radical Biology and Medicine*.

[128] Engels K., Knauer S. K., Loibl S. (2008). No signaling confers cytoprotectivity through the survivin network in ovarian carcinomas. *Cancer Research*.

[129] Yang H. L., Lin R. W., Rajendran P. (2019). Antrodia salmonea-induced oxidative stress abrogates Her-2 signaling cascade and enhanced apoptosis in ovarian carcinoma cells. *Journal of Cellular Physiology*.

[130] Ford C. E., Henry C., Llamosas E., Djordjevic A., Hacker N. (2016). Wnt signalling in gynaecological cancers: a future target for personalised medicine?. *Gynecologic Oncology*.

[131] McMellen A., Woodruff E. R., Corr B. R., Bitler B. G., Moroney M. R. (2020). Wnt signaling in gynecologic malignancies. *International Journal of Molecular Sciences*.

[132] Nguyen V. H. L., Hough R., Bernaudo S., Peng C. (2019). Wnt/*β*-catenin signalling in ovarian cancer: insights into its hyperactivation and function in tumorigenesis. *Journal of Ovarian Research*.

[133] Arend R. C., Londono-Joshi A. I., Straughn J. M., Buchsbaum D. J. (2013). The Wnt/*β*-catenin pathway in ovarian cancer: A review. *Gynecologic Oncology*.

[134] Goldsberry W. N., Meza-Perez S., Londoño A. I. (2020). Inhibiting Wnt ligand production for improved immune recognition in the ovarian tumor microenvironment. *Cancers*.

[135] Betella I., Turbitt W. J., Szul T. (2020). Wnt signaling modulator Dkk1 as an immunotherapeutic target in ovarian cancer. *Gynecologic Oncology*.

[136] Wu Y., Antony S., Meitzler J. L., Doroshow J. H. (2014). Molecular mechanisms underlying chronic inflammation-associated cancers. *Cancer Letters*.

[137] Teeuwssen M., Fodde R. (2019). Wnt signaling in ovarian cancer stemness, EMT, and therapy resistance. *Journal of Clinical Medicine*.

[138] Vallee A., Lecarpentier Y. (2018). Crosstalk between peroxisome proliferator-activated receptor gamma and the canonical WNT/*β*-Catenin pathway in chronic inflammation and oxidative stress during Carcinogenesis. *Frontiers in Immunology*.

[139] Yan X., Lyu T., Jia N., Yu Y., Hua K., Feng W. (2013). Huaier aqueous extract inhibits ovarian cancer cell motility via the AKT/GSK3*β*/*β*-Catenin pathway. *PLoS One*.

[140] Lili L. N., Matyunina L. V., Walker L. D., Wells S. L., Benigno B. B., McDonald J. F. (2013). Molecular profiling supports the role of epithelial-to-mesenchymal transition (EMT) in ovarian cancer metastasis. *Journal of Ovarian Research*.

[141] Davidson B., Trope C. G., Reich R. (2012). Epithelial-mesenchymal transition in ovarian carcinoma. *Frontiers in Oncology*.

[142] Wen J., Zhao Z., Huang L., Wang L., Miao Y., Wu J. (2020). IL-8 promotes cell migration through regulating EMT by activating the Wnt/*β*‐catenin pathway in ovarian cancer. *Journal of Cellular and Molecular Medicine*.

[143] Raghavan S., Mehta P., Xie Y., Lei Y. L., Mehta G. (2019). Ovarian cancer stem cells and macrophages reciprocally interact through the Wnt pathway to promote pro-tumoral and malignant phenotypes in 3D engineered microenvironments. *Journal for ImmunoTherapy of Cancer*.

[144] Ruan X., Liu A., Zhong M. (2019). Silencing LGR6 Attenuates Stemness and Chemoresistance via Inhibiting Wnt/*β*-Catenin Signaling in Ovarian Cancer. *Molecular Therapy - Oncolytics*.

[145] al-Alem L., Curry T. E. (2015). Ovarian cancer: involvement of the matrix metalloproteinases. *Reproduction*.

[146] Wall J. A., Meza-Perez S., Scalise C. B. (2021). Manipulating the Wnt/*β*-catenin signaling pathway to promote anti-tumor immune infiltration into the TME to sensitize ovarian cancer to ICB therapy. *Gynecologic Oncology*.

[147] Barghout S. H., Zepeda N., Xu Z., Steed H., Lee C. H., Fu Y. (2015). Elevated *β*-catenin activity contributes to carboplatin resistance in A2780cp ovarian cancer cells. *Biochemical and Biophysical Research Communications*.

[148] Sun Z., Xu S., Cai Q. (2020). Wnt/*β*-catenin agonist BIO alleviates cisplatin-induced nephrotoxicity without compromising its efficacy of anti-proliferation in ovarian cancer. *Life Sciences*.

[149] Groeneweg J. W., Foster R., Growdon W. B., Verheijen R. H., Rueda B. R. (2014). Notch signaling in serous ovarian cancer. *Journal of Ovarian Research*.

[150] Akbarzadeh M., Akbarzadeh S., Majidinia M. (2020). Targeting Notch signaling pathway as an effective strategy in overcoming drug resistance in ovarian cancer. *Pathology - Research and Practice*.

[151] Silva F., Felix A., Serpa J. (2016). Functional redundancy of the Notch pathway in ovarian cancer cell lines. *Oncology Letters*.

[152] Xie Q., Cheng Z., Chen X., Lobe C. G., Liu J. (2017). The role of Notch signalling in ovarian angiogenesis. *Journal of Ovarian Research*.

[153] Tzeng T. J., Cao L., Fu Y., Zeng H., Cheng W. H. (2014). Methylseleninic acid sensitizes Notch3-activated Ovca429 ovarian cancer cells to carboplatin. *PLoS One*.

[154] Bocchicchio S., Tesone M., Irusta G. (2019). Convergence of Wnt and Notch signaling controls ovarian cancer cell survival. *Journal of Cellular Physiology*.

[155] Canté-Barrett K., Holtzer L., van Ooijen H. (2020). A molecular test for quantifying functional Notch signaling pathway activity in human cancer. *Cancers*.

[156] Silwal-Pandit L., Langerod A., Borresen-Dale A. L. (2017). TP53Mutations in breast and ovarian cancer. *Cold Spring Harbor Perspectives in Medicine*.

[157] Nakamura M., Obata T., Daikoku T., Fujiwara H. (2019). The association and significance of P53 in gynecologic cancers: the potential of targeted therapy. *International Journal of Molecular Sciences*.

[158] Kim O., Park E. Y., Klinkebiel D. L. (2020). In vivo modeling of metastatic human high-grade serous ovarian cancer in mice. *PLoS Genetics*.

[159] Cordani M., Butera G., Pacchiana R. (2020). Mutant P53-associated molecular mechanisms of ROS regulation in cancer cells. *Biomolecules*.

[160] Mantovani F., Collavin L., Del Sal G. (2019). Mutant P53 as a guardian of the cancer cell. *Cell Death & Differentiation*.

[161] Liu X., Fan L., Lu C., Yin S., Hu H. (2020). Functional role of P53 in the regulation of chemical-induced oxidative stress. *Oxidative Medicine and Cellular Longevity*.

[162] Bai D. P., Zhang X. F., Zhang G. L., Huang Y. F., Gurunathan S. (2017). Zinc oxide nanoparticles induce apoptosis and autophagy in human ovarian cancer cells. *International Journal of Nanomedicine*.

[163] Maiti A. K. (2010). Gene network analysis of oxidative stress-mediated drug sensitivity in resistant ovarian carcinoma cells. *The Pharmacogenomics Journal*.

[164] Suh Y. A., Post S. M., Elizondo-Fraire A. C. (2011). Multiple stress signals activate mutant p53In vivo. *Cancer Research*.

[165] Padmanabhan A., Kaushik M., Niranjan R., Richards J. S., Ebright B., Venkatasubbu G. D. (2019). Zinc oxide nanoparticles induce oxidative and proteotoxic stress in ovarian cancer cells and trigger apoptosis independent of P53-mutation status. *Applied Surface Science*.

[166] Zeligs K. P., Neuman M. K., Annunziata C. M. (2016). Molecular pathways: the balance between cancer and the immune system challenges the therapeutic specificity of targeting nuclear factor-*Κ*b signaling for cancer treatment. *Clinical Cancer Research*.

[167] White K. L., Rider D. N., Kalli K. R., Knutson K. L., Jarvik G. P., Goode E. L. (2011). Genomics of the NF-*κ*B signaling pathway: hypothesized role in ovarian cancer. *Cancer Causes Control*.

[168] Puar Y. R., Shanmugam M. K., Fan L., Arfuso F., Sethi G., Tergaonkar V. (2018). Evidence for the involvement of the master transcription factor NF-*κ*B in cancer initiation and progression. *Biomedicines*.

[169] Harrington B. S., Annunziata C. M. (2019). NF-*κ*B signaling in ovarian cancer. *Cancers*.

[170] Kleinschmidt E. G., Miller N. L. G., Ozmadenci D. (2019). Rgnef promotes ovarian tumor progression and confers protection from oxidative stress. *Oncogene*.

[171] Kiningham K. K., Xu Y., Daosukho C., Popova B., Clair D. K. S. T. (2001). Nuclear factor *κ*B-dependent mechanisms coordinate the synergistic effect of PMA and cytokines on the induction of superoxide dismutase 2. *The Biochemical Journal*.

[172] Mortezaee K., Najafi M., Farhood B., Ahmadi A., Shabeeb D., Musa A. E. (2019). NF-*κ*B targeting for overcoming tumor resistance and normal tissues toxicity. *Journal of Cellular Physiology*.

[173] Gloire G., Piette J. (2009). Redox regulation of nuclear post-translational modifications during NF-*κ*B activation. *Antioxidants & Redox Signaling*.

[174] Pei H., Yang Y., Cui L. (2016). Bisdemethoxycurcumin inhibits ovarian cancer via reducing oxidative stress mediated MMPs expressions. *Scientific Reports*.

[175] Buelna-Chontal M., Zazueta C. (2013). Redox activation of Nrf2 & NF-*κ*B: a double end sword?. *Cellular Signalling*.

[176] Seeber L. M., Horrée N., Vooijs M. A. (2011). The role of hypoxia inducible factor-1alpha in gynecological cancer. *Critical Reviews in Oncology/Hematology*.

[177] Semenza G. L. (2002). HIF-1 and tumor progression: pathophysiology and therapeutics. *Trends in Molecular Medicine*.

[178] Galanis A., Pappa A., Giannakakis A., Lanitis E., Dangaj D., Sandaltzopoulos R. (2008). Reactive oxygen species and HIF-1 signalling in cancer. *Cancer Letters*.

[179] Ao Q., Su W., Guo S., Cai L., Huang L. (2015). SENP1 desensitizes hypoxic ovarian cancer cells to cisplatin by up- regulating HIF-1*α*. *Scientific Reports*.

[180] Zhang P., Liu Y., Feng Y., Gao S. (2016). Snail gene inhibited by hypoxia-inducible factor 1*α* (HIF-1*α*) in epithelial ovarian cancer. *International Journal of Immunopathology and Pharmacology*.

[181] Wang Y., Ma J., Shen H. (2014). Reactive oxygen species promote ovarian cancer progression via the HIF-1*α*/Lox/E-cadherin pathway. *Oncology Reports*.

[182] Zhao T., Zhao C., Zhou Y., Zheng J., Gao S., Lu Y. (2017). HIF-1*α*binding to AEG-1 promoter induced upregulated AEG-1 expression associated with metastasis in ovarian cancer. *Cancer Medicine*.

[183] Lu T., Tang J., Shrestha B. (2020). Up-regulation of hypoxia-inducible factor antisense as a novel approach to treat ovarian cancer. *Theranostics*.

[184] Nair D., Rådestad E., Khalkar P. (2018). Methylseleninic acid sensitizes ovarian cancer cells to T-cell mediated killing by decreasing PDL1 and VEGF levels. *Frontiers in Oncology*.

[185] Huang L., Ao Q., Zhang Q. (2010). Hypoxia induced paclitaxel resistance in human ovarian cancers via hypoxia-inducible factor 1*α*. *Journal of Cancer Research and Clinical Oncology*.

[186] Lindgren A., Anttila M., Rautiainen S. (2019). Dynamic contrast-enhanced perfusion parameters in ovarian cancer: good accuracy in identifying high HIF-1*α* expression. *PLoS One*.

[187] Jiang Y., Wang C., Zhou S. (2020). Targeting tumor microenvironment in ovarian cancer: premise and promise. *Biochimica et Biophysica Acta (BBA) - Reviews on Cancer*.

[188] Worzfeld T., Pogge von Strandmann E., Huber M. (2017). The unique molecular and cellular microenvironment of ovarian cancer. *Frontiers in Oncology*.

[189] Zhang B., Chen F., Xu Q. (2018). Revisiting ovarian cancer microenvironment: a friend or a foe?. *Protein Cell*.

[190] Nakao N., Kurokawa T., Nonami T., Tumurkhuu G., Koide N., Yokochi T. (2008). Hydrogen peroxide induces the production of tumor necrosis factor-*α* in RAW 264.7 macrophage cells via activation of P38 and stress-activated protein kinase. *Innate Immunity*.

[191] Blaser H., Dostert C., Mak T. W., Brenner D. (2016). TNF and ROS crosstalk in inflammation. *Trends in Cell Biology*.

[192] Rasool M., Malik A., Basit Ashraf M. A. (2016). Evaluation of matrix metalloproteinases, cytokines and their potential role in the development of ovarian cancer. *PLoS One*.

[193] Weiss J. M., Davies L. C., Karwan M. (2018). Itaconic acid mediates crosstalk between macrophage metabolism and peritoneal tumors. *Journal of Clinical Investigation*.

[194] Klink M., Jastrzembska K., Nowak M. (2008). Ovarian cancer cells modulate human blood neutrophils response to ActivationIn vitro. *Scandinavian Journal of Immunology*.

[195] Perego M., Tyurin V. A., Tyurina Y. Y. (2020). Reactivation of dormant tumor cells by modified lipids derived from stress-activated neutrophils. *Science Translational Medicine*.

[196] Zheng Z. M., Yang H. L., Lai Z. Z. (2020). Myeloid-derived suppressor cells in obstetrical and gynecological diseases. *American Journal of Reproductive Immunology*.

[197] Baert T., Vankerckhoven A., Riva M. (2019). Myeloid derived suppressor cells: key drivers of immunosuppression in ovarian cancer. *Frontiers in Immunology*.

[198] Okla K., Czerwonka A., Wawruszak A. (2019). Clinical relevance and immunosuppressive pattern of circulating and infiltrating subsets of myeloid-derived suppressor cells (MDSCs) in epithelial ovarian cancer. *Frontiers in Immunology*.

[199] Li X., Wang J., Wu W. (2020). Myeloid-derived suppressor cells promote epithelial ovarian cancer cell stemness by inducing the CSF2/P-STAT3 signalling pathway. *The FEBS Journal*.

[200] Ohl K., Tenbrock K. (2018). Reactive oxygen species as regulators of MDSC-mediated immune suppression. *Frontiers in Immunology*.

[201] Wei J., Zhang M., Zhou J. (2015). Myeloid-derived suppressor cells in major depression patients suppress T-cell responses through the production of reactive oxygen species. *Psychiatry Research*.

[202] Yang Y., Bazhin A. V., Werner J., Karakhanova S. (2013). Reactive oxygen species in the immune system. *International Reviews of Immunology*.

[203] Nagaraj S., Gupta K., Pisarev V. (2007). Altered recognition of antigen is a mechanism of Cd8+ T cell tolerance in cancer. *Nature Medicine*.

[204] Maj T., Wang W., Crespo J. (2017). Oxidative stress controls regulatory T cell apoptosis and suppressor activity and Pd-L1-blockade resistance in tumor. *Nature Immunology*.

[205] Battisti F., Napoletano C., Rahimi Koshkaki H. (2017). Tumor-derived microvesicles modulate antigen cross-processing via reactive oxygen species-mediated alkalinization of phagosomal compartment in dendritic cells. *Frontiers in Immunology*.

[206] Dionisi M., de Archangelis C., Battisti F. (2018). Tumor-derived microvesicles enhance cross-processing ability of clinical grade dendritic cells. *Frontiers in Immunology*.

[207] Mikuła-Pietrasik J., Uruski P., Szubert S. (2017). Malignant ascites determine the transmesothelial invasion of ovarian cancer cells. *The International Journal of Biochemistry & Cell Biology*.

[208] Yang W., Toffa S. E., Lohn J. W., Seifalian A. M., Winslet M. C. (2005). Malignant ascites increases the antioxidant ability of human ovarian (SKOV-3) and gastric adenocarcinoma (KATO-III) cells. *Gynecologic Oncology*.

[209] Worley B. L., Kim Y. S., Mardini J. (2019). GPx3 supports ovarian cancer progression by manipulating the extracellular redox environment. *Redox Biology*.

[210] Klomsiri C., Rogers L. A. C., Soito L. (2014). Endosomal H_2_O_2_ production leads to localized cysteine sulfenic acid formation on proteins during lysophosphatidic acid-mediated cell signaling. *Free Radical Biology and Medicine*.

[211] Saunders J. A., Rogers L. C., Klomsiri C., Poole L. B., Daniel L. W. (2010). Reactive oxygen species mediate lysophosphatidic acid induced signaling in ovarian cancer cells. *Free Radical Biology and Medicine*.

[212] Rogers L. C., Davis R. R., Said N., Hollis T., Daniel L. W. (2018). Blocking LPA-dependent signaling increases ovarian cancer cell death in response to chemotherapy. *Redox Biology*.

[213] Shao C., Yang F., Miao S. (2018). Role of hypoxia-induced exosomes in tumor biology. *Molecular Cancer*.

[214] Rogalska A., Gajek A., Lukawska M., Oszczapowicz I., Marczak A. (2018). Novel oxazolinoanthracyclines as tumor cell growth inhibitors-contribution of autophagy and apoptosis in solid tumor cells death. *PLoS One*.

[215] Yang H., Villani R. M., Wang H. (2018). The role of cellular reactive oxygen species in cancer chemotherapy. *Journal of Experimental & Clinical Cancer Research*.

[216] Zhao F., Hong X., Li D., Wei Z., Ci X., Zhang S. (2021). Diosmetin induces apoptosis in ovarian cancer cells by activating reactive oxygen species and inhibiting the Nrf2 pathway. *Medical Oncology*.

[217] AlBasher G., AlKahtane A. A., Alarifi S. (2019). Methotrexate-induced apoptosis in human ovarian adenocarcinoma SKOV-3 cells via ROS-mediated Bax/Bcl-2-Cyt-C release cascading. *OncoTargets and Therapy*.

[218] Hou D., Liu Z., Xu X. (2018). Increased oxidative stress mediates the antitumor effect of PARP inhibition in ovarian cancer. *Redox Biology*.

[219] Ai Z., Lu Y., Qiu S., Fan Z. (2016). Overcoming cisplatin resistance of ovarian cancer cells by targeting HIF-1-regulated cancer metabolism. *Cancer Letters*.

[220] Ahmad T., Suzuki Y. J. (2019). Juglone in oxidative stress and cell signaling. *Antioxidants*.

[221] Fang F., Zhao L. Z., Li Q. (2016). Effects of juglone on oxidative stress and apoptosis of ovarian cancer SKOV3 cells. *Chinese Journal of Pathophysiology*.

[222] Cucci M. A., Grattarola M., Dianzani C. (2020). Ailanthone increases oxidative stress in CDDP-resistant ovarian and bladder cancer cells by inhibiting of Nrf2 and YAP expression through a post- translational mechanism. *Free Radical Biology and Medicine*.

[223] Benot-Dominguez R., Tupone M. G., Castelli V. (2021). Olive leaf extract impairs mitochondria by pro-oxidant activity in MDA-MB-231 and OVCAR-3 cancer cells. *Biomedicine & Pharmacotherapy*.

[224] Taparia S. S., Khanna A. (2016). Procyanidin-rich extract of natural cocoa powder causes ROS-mediated caspase-3 dependent apoptosis and reduction of pro-MMP-2 in epithelial ovarian carcinoma cell lines. *Biomedicine & Pharmacotherapy*.

[225] Seino M., Okada M., Shibuya K. (2015). Differential contribution of ROS to resveratrol-induced cell death and loss of self-renewal capacity of ovarian cancer stem cells. *Anticancer Research*.

[226] Kim T. H., Park J. H., Woo J. S. (2019). Resveratrol induces cell death through ROS-dependent downregulation of Notch1/Pten/Akt signaling in ovarian cancer cells. *Molecular Medicine Reports*.

[227] Mikuła-Pietrasik J., Sosińska P., Murias M. (2015). High Potency of a Novel Resveratrol Derivative, 3,3′,4,4′-Tetrahydroxy-trans- stilbene, against Ovarian Cancer Is Associated with an Oxidative Stress- Mediated Imbalance between DNA Damage Accumulation and Repair. *Oxidative Medicine and Cellular Longevity*.

[228] Sahai R., Bhattacharjee A., Shukla V. N. (2020). Gedunin isolated from the mangrove plant Xylocarpus granatum exerts its anti-proliferative activity in ovarian cancer cells through G2/M-phase arrest and oxidative stress-mediated intrinsic apoptosis. *Apoptosis*.

[229] Hsieh T. C., Wu J. M. (2011). Suppression of proliferation and oxidative stress by extracts of Ganoderma lucidum in the ovarian cancer cell line OVCAR-3. *International Journal of Molecular Medicine*.

[230] Liu S. S., Zhou Y. Q. (2016). Research progress of nanotechnology in ovarian cancer. *Medical Review*.

[231] Santos I., Ramos C., Mendes C. (2019). Targeting glutathione and cystathionine *Β*-synthase in ovarian cancer treatment by selenium-chrysin polyurea dendrimer nanoformulation. *Nutrients*.

[232] Niu W., Wang J., Wang Q., Shen J. (2020). Celastrol loaded nanoparticles with ROS-response and ROS-inducer for the treatment of ovarian cancer. *Frontiers in Chemistry*.

[233] Elejalde E., Villaran M. C., Alonso R. M. (2021). Grape polyphenols supplementation for exercise-induced oxidative stress. *Journal of the International Society of Sports Nutrition*.

[234] Poprac P., Jomova K., Simunkova M., Kollar V., Rhodes C. J., Valko M. (2017). Targeting free radicals in oxidative stress-related human diseases. *Trends in Pharmacological Sciences*.

[235] Huang T. T., Lampert E. J., Coots C., Lee J. M. (2020). Targeting the PI3K pathway and DNA damage response as a therapeutic strategy in ovarian cancer. *Cancer Treatment Reviews*.

[236] Mabuchi S., Hisamatsu T., Kimura T. (2011). Targeting mTOR signaling pathway in ovarian cancer. *Current Medicinal Chemistry*.

[237] Wang J., Jin L., Li X. (2013). Gossypol induces apoptosis in ovarian cancer cells through oxidative stress. *Molecular BioSystems*.

[238] Hou D., Xu G., Zhang C. (2017). Berberine induces oxidative DNA damage and impairs homologous recombination repair in ovarian cancer cells to confer increased sensitivity to PARP inhibition. *Cell Death & Disease*.

[239] Li R., Xiao J., Tang S. (2020). Cucurbitacin I induces apoptosis in ovarian cancer cells through oxidative stress and the P190b-Rac1 signaling axis. *Molecular Medicine Reports*.

[240] Mbemi A. T., Sims J. N., Yedjou C. G., Noubissi F. K., Gomez C. R., Tchounwou P. B. (2020). Vernonia calvoana shows promise towards the treatment of ovarian cancer. *International Journal of Molecular Sciences*.

[241] Tavsan Z., Kayali H. A. (2019). Flavonoids showed anticancer effects on the ovarian cancer cells: involvement of reactive oxygen species, apoptosis, cell cycle and invasion. *Biomedicine & Pharmacotherapy*.

[242] Wang Y. Y., Chen Y. K., Hu S. C. (2017). Cyt-Rx20 inhibits ovarian cancer cells in vitro and in vivo through oxidative stress-induced DNA damage and cell apoptosis. *Cancer Chemotherapy and Pharmacology*.

[243] Park S., Lim W., Jeong W., Bazer F. W., Lee D., Song G. (2018). Sideroxylin (Callistemon lanceolatus) suppressed cell proliferation and increased apoptosis in ovarian cancer cells accompanied by mitochondrial dysfunction, the generation of reactive oxygen species, and an increase of lipid peroxidation. *Journal of Cellular Physiology*.

[244] Xue J., Li R., Zhao X. (2018). Morusin induces paraptosis-like cell death through mitochondrial calcium overload and dysfunction in epithelial ovarian cancer. *Chemico-Biological Interactions*.

